# Strategies to enhance rational use of antibiotics in hospital: a guideline by the German Society for Infectious Diseases

**DOI:** 10.1007/s15010-016-0885-z

**Published:** 2016-04-11

**Authors:** K. de With, F. Allerberger, S. Amann, P. Apfalter, H.-R. Brodt, T. Eckmanns, M. Fellhauer, H. K. Geiss, O. Janata, R. Krause, S. Lemmen, E. Meyer, H. Mittermayer, U. Porsche, E. Presterl, S. Reuter, B. Sinha, R. Strauß, A. Wechsler-Fördös, C. Wenisch, W. V. Kern

**Affiliations:** Division of Infectious Diseases, University Hospital Carl Gustav Carus at the TU Dresden, Fetscherstr. 74, 01307 Dresden, Germany; Division Public Health, Austrian Agency for Health and Food Safety (AGES), Vienna, Austria; Hospital Pharmacy, Munich Municipal Hospital, Munich, Germany; Institute for Hygiene, Microbiology and Tropical Medicine (IHMT), National Reference Centre for Nosocomial Infections and Antimicrobial Resistance, Elisabethinen Hospital Linz, Linz, Austria; Department of Infectious Disease Medical Clinic II, Goethe-University Frankfurt, Frankfurt, Germany; Department for Infectious Disease Epidemiology, Robert Koch Institute, Berlin, Germany; Hospital Pharmacy, Schwarzwald-Baar Hospital, Villingen-Schwenningen, Germany; Department of Hospital Epidemiology and Infectiology, Sana Kliniken AG, Ismaning, Germany; Department for Hygiene and Infection Control, Danube Hospital, Vienna, Austria; Section of Infectious Diseases and Tropical Medicine, Medical University of Graz, Graz, Austria; Division of Infection Control and Infectious Diseases, University Hospital RWTH Aachen, Aachen, Germany; Institute of Hygiene and Environmental Medicine, Charité, University Medicine Berlin, Berlin, Germany; Department for Clinical Pharmacy and Drug Information, Landesapotheke, Landeskliniken Salzburg (SALK), Salzburg, Austria; Department of Infection Control and Hospital Epidemiology, Medical University of Vienna, Vienna, Austria; Clinic for General Internal Medicine, Infectious Diseases, Pneumology and Osteology, Klinikum Leverkusen, Leverkusen, Germany; Department of Medical Microbiology and Infection Prevention, University of Groningen, University Medical Center Groningen, Groningen, The Netherlands; Department of Medicine 1, Gastroenterology, Pneumology and Endocrinology, University Hospital Erlangen, Erlangen, Germany; Department of Antibiotics and Infection Control, Krankenanstalt Rudolfstiftung, Vienna, Austria; Medical Department of Infection and Tropical Medicine, Kaiser Franz Josef Hospital, Vienna, Austria; Division of Infectious Diseases, Department of Medicine, Freiburg University Medical Center, Freiburg, Germany

**Keywords:** Antibiotic stewardship, ABS, Guideline, Antimicrobial resistance, Quality of care, Rational use

## Abstract

**Introduction:**

In the time of increasing resistance and paucity of new drug development there is a growing need for strategies to enhance rational use of antibiotics in German and Austrian hospitals. An evidence-based guideline on recommendations for implementation of antibiotic stewardship (ABS) programmes was developed by the German Society for Infectious Diseases in association with the following societies, associations and institutions: German Society of Hospital Pharmacists, German Society for Hygiene and Microbiology, Paul Ehrlich Society for Chemotherapy, The Austrian Association of Hospital Pharmacists, Austrian Society for Infectious Diseases and Tropical Medicine, Austrian Society for Antimicrobial Chemotherapy, Robert Koch Institute.

**Materials and methods:**

A structured literature research was performed in the databases EMBASE, BIOSIS, MEDLINE and *The Cochrane Library* from January 2006 to November 2010 with an update to April 2012 (MEDLINE and *The Cochrane Library*). The grading of recommendations in relation to their evidence is according to the AWMF Guidance Manual and Rules for Guideline Development.

**Conclusion:**

The guideline provides the grounds for rational use of antibiotics in hospital to counteract antimicrobial resistance and to improve the quality of care of patients with infections by maximising clinical outcomes while minimising toxicity. Requirements for a successful implementation of ABS programmes as well as core and supplemental ABS strategies are outlined. The German version of the guideline was published by the German Association of the Scientific Medical Societies (AWMF) in December 2013.

## Introduction and aims of the guideline

The dramatic increase in antibiotic resistance seen in many areas and regions combined with the paucity of new drug development more than ever calls for prudent, controlled, and appropriate use of antiinfectives in all areas of medicine. This affects almost all disciplines and medical specialties. The density of antiinfective treatment—with all its implications for cost, toxicity, the emergence of resistance and recommendations on diagnosis and follow-up, as well as recommendations on further therapy in the outpatient setting—is so high, in particular in the hospital sector, that safety and quality assurance processes will no longer succeed without a panel of experts and strategic discussions. Following a first position paper of the European Commission in 2001, in a second report on “Prudent use of antimicrobial agents in human medicine” published in 2010, EU Member States were recommended to establish or enhance surveillance systems for antibiotic resistance and antibiotic consumption. Particular importance gains this recommendation in Germany in light of the amendment of the Infection Protection Act [Infektionsschutzgesetz (IfSG), especially §4 and §23] in July 2011. The Act not only stipulates collection of data on antibiotic consumption, pathogenic microorganisms and resistance, it also requires that data on antibiotic consumption be assessed taking into account the local resistance situation, and that appropriate conclusions be drawn regarding the use of antibiotics. Furthermore, that the necessary adjustments to antibiotic consumption be communicated to staff and implemented (IfSG § 23 paragraph 4). Antibiotic stewardship (ABS) programmes should and can assume this responsibility in combination with policies and programmes for infection prevention. The aim of ABS programmes in hospital is to continuously improve the quality of antiinfective prescribing with regard to agent selection, dosing, administration and duration of treatment in order to maximise clinical outcomes while minimising toxicity to the patient as well as the emergence of resistance and costs.

Many reviews published since 2005 [1–12] on antibiotic stewardship describe the requirements and elements needed to institutionalise this type of programme in hospitals. More recent publications also detail use of these programmes in intensive care units [13–15], paediatrics [16–18] or small community hospitals [19–21]. A relatively new systematic review on ABS activities in critical care medicine, assesses 24 studies between 1996 and 2010, among them six methodologically ambitious investigations [15]. These were projects limiting cephalosporin use to minimise the emergence of resistance, studies on the implementation of computerised decision support systems and infectious diseases consultation services, as well as introduction of new guidelines for therapy and prophylaxis. The review provides good insight into the effects of various ABS strategies on consumption, costs and resistance, as demonstrated in recent years by a number of other original papers. Most studies show a 10–40 % reduction in antiinfective drug use, shorter treatment duration and cost reduction. Programmes that were active for longer than 6 months were also associated with an improvement in resistance rates depending on the drug–pathogen combination [15]. A recent Cochrane review of 2013 (89 studies up to 2007) reached a similar conclusion. The review shows that the effect of interventions (e.g. antimicrobial restriction) is usually delayed (6 months) in respect of microbiological endpoints (e.g. antibiotic resistance); however, a prompt effect (frequently as soon as 1 month) is noted with regard to prescribing endpoints. According to the meta-analysis of methodologically robust studies (including randomised and controlled before-after studies with interrupted time-series analyses), professional interventions to reduce excessive antiinfective prescribing are successful in minimising the emergence of resistance and reducing hospital-acquired infections, as well as in improving individual treatment outcomes [22].

The two most recent reviews mentioned demonstrate the importance of ABS programmes and rational prescribing strategies in terms of minimising resistance. Instituting ABS programmes to save costs is not any more the driving factor, although this aspect is still important. An analysis published in 2012 on the cost-effectiveness of an ABS programme initiated at a University Hospital in Maryland, USA, showed interesting results. Over a period of 3 years, gross savings of roughly USD 3 million were realised, i.e. approximately USD 1 million/year. This was opposed by expenses of roughly USD 200,000/year to finance the programme (personnel costs). Although yearly cost savings dropped to approximately USD 400,000 p.a. over the full 7-year term of the ABS programme, it nevertheless remained cost-effective and delivered net savings with only one full-time personnel per 500 beds (infectious diseases physician, pharmacist, IT specialist). When the programme was discontinued after 7 years, antiinfective costs rapidly rose by around USD 2 million during the subsequent 2 years [23]. Cost–benefit analyses performed in other more recent pharmacoeconomic investigations of ABS programmes are no longer limited to the potential “savings” achieved in drug and material costs; rather they show that adequate antiinfective therapy is associated with lower mortality, shorter length of hospital stay and duration of treatment, and that it can reduce the overall cost of treatment and improve patient safety [24].

This guideline recommends and outlines the requirements and main elements of ABS programmes with which the above objectives can be achieved. The recommendations are based on a systematic evaluation of many new observational and interventional studies with clinical and microbiological endpoints, as well as the endpoints antiinfective prescribing and costs, which were mainly conducted in adult patients in acute-care hospitals. The available literature was compiled based on the guideline published by two American societies (IDSA, SHEA) focusing on the development of facility-specific ABS programmes (“Guidelines for Developing an Institutional Program to Enhance Antimicrobial Stewardship”) as well as on a Cochrane Review by Davey et al. from 2005 on “Interventions to improve antibiotic prescribing practices for hospital inpatients (Review)”, taking into account its update (2013) [2, 6, 22, 25]. Further literature was systematically searched until 15 April 2012 and evaluated. For details see the methodology report published online (http://www.awmf.org). Although some of the recommendations are not new in content, they altogether draw on much better study evidence and a greater number of examples for successful programmes. The recommendations were derived by consensus by the guideline development group based on review of the literature, taking into account relevance, evidence, applicability and practicability in German and Austrian acute-care hospitals. Key challenges are the current trends in multidrug-resistant pathogens (VRE, multidrug-resistant Gram-negative bacteria) and *Clostridium difficile* in Germany and Austria, the lack of skilled personnel—especially infectious diseases physicians—and limited experience with well-functioning infectious disease consultation services established elsewhere, increasing cost pressure in hospitals and outsourcing of microbiological diagnostics.

For the purpose of safety and quality assurance, it is recommended to use a selection of indicators from a catalogue developed and agreed upon by members of the guideline development group and users in Germany. Further experience with validation especially of process indicators as well as international experience gained in particularly France, England and Scotland on use of such indicators for internal and external quality assurance should be taken into account.

## Summary of recommendations

### Requirements

#### Availability of a team of ABS experts

For effective implementation of ABS programmes, it is essential that a multidisciplinary team should be instructed by the hospital administration and allocated with adequate resources to draw up guidelines derived by consensus with the users for the treatment of infectious diseases and to ensure their implementation through ABS strategies **(A)**.

The team should consist of at least one infectious diseases physician (or clinician with infectious diseases training) and an experienced clinical pharmacist/hospital pharmacist, as well as a specialist in microbiology, virology and infection epidemiology being responsible for laboratory diagnostic and microbiological consultation; furthermore, the physician locally responsible for infection control. The team members should either have appropriate training in antibiotic stewardship or already be sufficiently experienced **(A)**.

The team will receive the support and collaboration of the hospital administration, and activities within the ABS programme should be compensated with a minimum of one full-time equivalent (FTE) of 0.5 per 250 beds **(A)**. There should be good collaboration between the Therapeutics and Drugs Committee, Hospital Infection Control Committee, pharmacy and representatives of clinical divisions/departments (ABS representatives), for which purpose the team should issue its own Rules of Procedure **(A)**.

Significance in practice:

- ABS programmes should be instituted facility-wide which necessitates a multidisciplinary team with the competence for interdisciplinary cooperation.
- Infectious diseases specialists serving as consultants improve the clinical outcome of patients with infections, and ensure the quality of drug prescribing.
- Clinical pharmacists improve the quality of drug prescribing (e.g. dosing and drug application, avoidance of adverse drug events).
- Microbiologists facilitate high-grade infection medicine by ensuring the quality of microbiological diagnostics and preanalytics, and by expertly evaluating and conveying microbial culture results.
- Various ABS programmes describe an FTE of 0.5 per 250 beds as being the minimum staff resources necessary to cost-effectively conduct an ABS programme.

#### Availability of surveillance data on pathogens, resistance, and antimicrobial consumption

##### Pathogens and resistance

Antimicrobial susceptibility data on major pathogens should be available and accessible at least yearly on a hospital-wide level and separately for general and intensive care units, or department-specific, as the case may be. Data on primary isolates should be shown by pathogen and type of specimen, e.g. blood, urine, miscellaneous samples. Culture results from screening tests should be shown separately. Susceptibility rates should indicate the number of isolates tested. Infection rates should relate consistently to a single denominator (e.g. patient-days/number of cases). Participation in an established surveillance system is recommended **(A)**.

Significance in practice:

- Conducting an additional material analysis (e.g. number of blood culture sets per patient or 1000 patient-days, number of urine cultures per patient, number of catheter-associated urine cultures, etc.) also with regard to material quality and positivity/contamination rates (e.g. blood cultures) can be useful.
- Whether the susceptibility rates of pathogens should be limited to agents listed on the hospital formulary should be discussed within the team.
- The amendment of the Infection Protection Act (Infektionsschutzgesetz, IfSG) (reporting, documentation) is mandatory.

##### Antimicrobial consumption

Data on antimicrobial consumption, expressed as use density (daily doses per 100 patient-days) should be collected at least annually or preferably quarterly and are generally reported by the pharmacist. Data are reported institution-wide, at the ward level as well as for individual (speciality) departments. On demand, data should be broken down to the agent level and should be provided to the ABS team. Participation in an established surveillance system is recommended **(A)**.

Point prevalence surveys should be conducted for systematic quantitative and qualitative assessment of antiinfective use, and, if required should be reevaluated short-term **(A)**. Antiinfective use data are collected at the patient level which allows to assess prescribing quality based on indication and type of infection, and to recognise the need for targeted ABS strategies. Access to patient-level data ought to be guaranteed.

Significance in practice:

- Use density should be presented by antibiotic class and not only by individual agent.
- Reporting consumption data and antiinfective costs ranked by individual agent or class (e.g. top 5 or 10) is also reasonable.
- Point prevalence surveys are a simple tool to examine process quality.
- The amendment of the Infection Protection Act (Infektionsschutzgesetz, IfSG) (reporting, documentation) must be observed.

### ABS core strategies

#### Application of local treatment guidelines/pathways, hospital antiinfective formulary, formulary restrictions and approval requirements

Developing and updating local treatment guidelines, clinical pathways, and an antiinfective formulary is one of the ABS team’s chief responsibilities. The antiinfective formulary should be based on national and international guidelines as well as on the local/regional pathogen and resistance patterns, and possibly drug costs. Drugs on the antiinfective formulary should be categorised according to recommended versus reserve or special compounds. In addition, these should be tagged with special prescription status and be subject to approval and preauthorisation requirements. The antiinfective formulary is updated at least yearly based on therapy guidelines and whenever necessary and approved by the Therapeutics and Drugs Committee **(A)**.

Adherence to guidelines regarding substance selection, dosing, route and duration of treatment may improve clinical outcome in terms of mortality, as well as treatment duration and length of hospital stay. To ensure adherence, users should be involved in developing the guidelines and be educated through audits of antiinfective use or antiinfective point-of-care chart reviews **(A)**.

Individualising antiinfective prescriptions with or without special approval requirements improves targeted therapy and reduces inappropriate treatment. Various possibilities for implementation have been described and should be used, from simple antimicrobial order forms to highly differentiated antiinfective request forms that may be subject to specific time limits or limited to certain hospital areas **(A)**. Guideline-based antiinfective drug use or use of individual defined substances can be controlled by this means, thus minimising consumption, costs and adverse drug events.

Restricting whole substance classes can—by shifting to an alternative substance—prove to be an effective strategy for controlling nosocomial infections and the development of critical resistance levels; accordingly, antiinfective restriction ought to be targeted **(B)**. At the same time, routine surveillance of antibiotic consumption and locally prevalent pathogens and their susceptibility patterns should be performed to detect possible adverse effects of the strategy in time **(A)**.

Significance in practice:

- Local guidelines serve quality assurance and are a core strategy of every ABS programme.
- The antiinfective formulary is a useful ABS tool especially in small and medium-sized hospitals.
- Clinical pathways are rarely employed, can, however, be very helpful in the emergency room.
- Special order forms are highly effective ABS tools. Efforts should be undertaken to foster acceptance by prescribers.
- Restrictions on use to control resistance and nosocomial infections are frequently only temporarily effective. They should be time-restricted by the ABS team and reconsidered depending on the effects.

#### Design and implementation of education, training and information

Targeted education, training and information are essential elements of any ABS programme. They provide the foundation of knowledge needed to promote more rational use of antibiotics and reasonable microbiological diagnostic, and to improve acceptance of ABS programmes. They have the objective of optimising the therapeutic and diagnostic management of patients with infection through greater adherence to recommendations. They should preferably take place as an active training measure rather than in the form of passive communication of information **(A)**.

Education, training and information in different formats and on various topics should be offered repeatedly as they are not sustainable as a one-off measure. They should be organised in agreement and integration with local ABS programmes **(A)**.

Education, training and information should be independent of commercial interests, whereby the hospital administration is responsible for implementing and financing the measures **(A)**.

Significance in practice:

- The target group for local training and educational sessions should be clearly defined.
- The handling of conflicts of interest should be laid down in writing (Rules of Procedure) by the ABS team.
- Informative meetings and educational/training sessions should give special attention to a critical evaluation of published study results.

#### Conducting proactive audits of antiinfective use

Proactive on-site audits of antiinfective use in the context of antiinfective point-of-care chart reviews are important elements of ABS programmes and should be performed routinely by the ABS team **(A)**. They enhance compliance with guidelines or clinical pathways, improve outcome in patients with infection and improve the quality of prescribing with regard to indication, choice of agent, dosing, dosing interval, administration route and treatment duration.

Depending on the problem and treatment target, besides point prevalence studies, agent-, indication- and/or diagnosis-related audits of antiinfective use should be conducted within the scope of regular antiinfective point-of-care chart reviews hospital-wide or at the unit level, whereby quality indicators should preferably be applied **(A)**.

Results should be fed back in direct interaction with the prescribing physicians and discussed with them **(A)**.

Significance in practice:

- Performing proactive audit of antiinfective use with review and feedback is time-consuming; it does, however, promote interdisciplinary collaboration.
- Antiinfective point-of-care chart reviews can increase the number of treatments complying with guidelines and thus substantially improve process quality.

#### Quality indicators

ABS programmes should be integrated within the hospital’s quality management. Content overlaps with the Therapeutics and Drugs Committee (drug safety) and Hospital Infection Control Committee (prevention of nosocomial infection) is useful and desired. Appropriate quality indicators to measure prescription practice (process measure), emergence of resistance or trend in consumption (outcome measure) and structure ought to be set and applied in every ABS programme **(B)**. At least three indicators measuring structural quality and at least three indicators measuring process quality should be set regularly **(A)**.

Significance in practice:

- Quality indicators are used to evaluate the progress of an ABS programme.
- Quality indicators help to recognise hospital areas which will benefit from the implementation of targeted and intensive ABS measures.

### Supplemental ABS strategies

#### Special programmes for treatment optimisation

##### De-escalation

A key aspect of supplemental measures is to streamline treatment after initial empirical broad-spectrum therapy and conversion from empirical to targeted therapy. This ought to be done based on clinical criteria as well as microbiology results or other diagnostic findings. De-escalation measures ought to preferably be performed at the patient level in the context of antiinfective point-of-care chart reviews and proactive audits of antiinfective drug use **(B)**. Programmes promoting antiinfective de-escalation are expected to, by reducing antibiotic load, impact beneficially on the emergence of resistance, the prevention of secondary infections, cost levels and adverse drug reactions **(B)**.

Significance in practice:

- De-escalation includes conversion from an empirical combination therapy to targeted monotherapy based on knowledge of the microorganism isolated, susceptibility and infectious disease.
- De-escalation should be initiated early on (after 48–72 h), which also includes discontinuation of initial therapy if diagnosis is not secured. Observational studies show that this strategy is not adopted in 20–60 % of cases.
- De-escalation programmes should point out that depending on the exact diagnosis in some cases instead of de-escalation, escalation may in fact be necessary.

##### Duration of treatment

It is possible to shorten the duration of antiinfective treatment for many indications (e.g. perioperative antibiotic prophylaxis) and this is recommended wherever backed by good studies and evidence. The ABS team should utilise local guidelines and antiinfective point-of-care chart reviews to draw attention to the excessive duration of treatment frequently encountered in practice. The ABS team should define the duration of treatment recommended as a rule, since this is expected to impact substantially on antiinfective drug use, side effects and costs **(A)**. Use of biomarkers such as Procalcitonin may be useful for controlling the duration of treatment in cases where there is clinical uncertainty. As a result, the number of days of antibiotic therapy can be reduced and under certain circumstances costs can be cut **(C)**.

Significance in practice:

- Shortening the duration of treatment appropriately reduces the density of antiinfective use without compromising clinical outcome or costs, it also minimises the emergence of resistance by decreasing selection pressure.
- The duration of treatment is well established for a number of indications, e.g. pneumonia, endocarditis, perioperative antibiotic prophylaxis. Therapy, thus, only needs to be individualised and extended in certain cases.

##### Parenteral-to-oral conversion

If sufficient bioavailability is assured, and if the patient’s condition allows, therapy should be switched from parenteral to oral antibiotic application **(A)**. This measure reduces the length of hospital stay and the risk of line-related adverse events. Furthermore, it leads to a reduction in the total cost of treatment. Implementation of programmes allowing parenteral-to-oral conversion of antimicrobial agents at the institutional level ought to be facilitated by developing clinical criteria and through explicit designation in institutional guidelines or clinical pathways **(B)**.

Significance in practice:

- Switch to oral therapy should be assessed on day 3–4 of parenteral antiinfective therapy.
- Switching to oral therapy not only results in direct cost savings (antiinfective agents, supplies, nursing time) and lowers risk of line infections, it also increases the patient’s mobility.

##### Dose optimisation

Adequate adjustment and optimisation of the dose and dosing interval is essential for effective, safe and responsible administration of antiinfective therapy, and an important part of ABS programmes. Besides individual patient factors, optimal dosing of antiinfectives should take into account the nature and severity of illness, the causative microorganism, concomitant medications, as well as the pharmacokinetics and pharmacodynamics of the agents prescribed. Strategies to optimise dosing in ABS programmes should include assessment of organ function for drug dose adjustment in order to avoid adverse drug events and unwanted drug interactions **(A)**.

Furthermore, optimising the dosing interval and duration of infusion is recommended in particular in critically ill patients, best by employing a therapeutic drug monitoring (TDM) scheme; appropriate consented local institutional guidelines should be available and up to date **(B)**.

Significance in practice:

- Prolonged infusion of beta-lactams (taking into account physico-chemical stability) is reasonable and recommended particularly in critically ill patients.
- TDM can avoid under-/over-dosing and minimise organ toxicity.
- Programmes for doses optimisation are cost-effective.

##### Scheduled switch of antimicrobials

So-called “Cycling” programmes, which involve periodically removing a specific antimicrobial drug or an antimicrobial drug class as the standard recommended therapy and later reintroducing it (periodic scheduled rotation), are not suitable as a strategy to reverse critical emergence of resistance or to control nosocomial outbreaks with multiple resistant pathogens and, as such, should not be used as a strategy to do so **(A)**.

Strategic rotation of specific antimicrobial drugs or antimicrobial drug classes ought to be undertaken to limit the selective pressures and to achieve a reduction of infectious microorganisms or microorganisms displaying specific resistance properties for a certain time **(B)**. There is evidence to suggest that a balanced use of different antimicrobial drugs or antimicrobial drug classes (so-called “mixing”) can minimise the emergence of resistance. In both cases, routine surveillance of antimicrobial drug use and resistance should be performed **(A)**.

Significance in practice:

- Strategic rotation of specific antimicrobials or antimicrobial classes should be planned by the ABS team in consultation with the facility’s infection control team and the microbiology department. Continuous surveillance of pathogens, resistance patterns and consumption is imperative.
- Guidelines and antiinfective formularies that recommend predominant use of fluoroquinolones or third-generation cephalosporins should be considered as critical.

#### Special rules for communication of microbiology results

The quality of microbiology diagnostics depends crucially on compliance with guidelines on procedures in the preanalytical phase. Expert consensus recommends that deviations from protocol ought to be reported and the reasons for rejecting the samples stated **(B)**.

Technical progress and up-to-date molecular diagnostic methods for rapid pathogen detection should be used if they improve the quality of care and/or substantially improve identification and epidemiologic investigation of local outbreaks **(A)**.

Positive blood culture findings, interim microscopic findings, results of rapid testing results and rapid susceptibility testing should be communicated promptly to the physician **(A)**.

Antibiograms ought to adhere to local guidelines with respect to antimicrobial use and diagnostic findings, be presented selectively in agreement with the ABS team, and, if need be, include relevant interpretative comments. This procedure aids selection of a targeted, guideline-based antibiotic regimen **(B)**.

The microbiology laboratory is responsible for the timely identification of critical trends in antimicrobial resistance and prompt communication of observations to the ABS team and the physicians responsible for infection control **(A)**. This way, the clinical and epidemiological significance of the observations can be defined at an early stage.

Significance in practice:

- Molecular diagnostic methods can expedite pathogen specification.
- Selective reporting of susceptibility results with respect to choice and number of antimicrobial agents, and comments on daily treatment costs, route of administration, hospital formulary drug, resistance mechanisms supports adherence to local guidelines.

#### Special rules for management of patients with multidrug-resistant microorganisms and *C. difficile*

ABS strategies should be used to prevent infection with *C.difficile***(A)**. Restricting use of certain antimicrobial drugs or substitution of antimicrobial drug classes (e.g. penicillin for cephalosporins or fluoroquinolones) can considerably reduce the incidence of *C.difficile* infection. Infection prevention and control strategies are frequently also applied at the same time; however, they have less impact on the *C. difficile* incidence than in the epidemiology of MRSA or VRE.

Targeted ABS strategies are to varying degrees also effective in reducing multidrug resistant Gram-negative bacteria, particularly ESBL-producing microorganisms, MRSA and VRE, and ought to be specifically applied here too **(B)**. In case of high prevalence of multidrug-resistant microorganisms, recommendations on diagnostic tests, evaluation of findings and treatment, as well as infection control management should be coordinated immediately and disseminated locally **(A)**.

Routine surveillance of antimicrobial consumption and antimicrobial susceptibility data should be performed **(A)** to avoid indiscriminate compensatory use of other antimicrobial drug classes, since this can promote the unintentional and uncontrolled emergence of resistance.

Significance in practice:

- Reducing consumption of cephalosporins and/or fluoroquinolones or substituting them for penicillin may reduce the frequency of *C. difficile* infection and possibly also have a beneficial effect on the incidence of infections caused by multidrug-resistant pathogens.

#### Computerised information technology

The ABS team should be supported by novel information and communication technology in the implementation of ABS programmes. Local treatment guidelines, the antiinfective formulary, and other ABS documents should be available electronically **(A)**.

Electronic prescribing tools with and without linkage to electronic preauthorisation solutions, to ABS documents or to active communication of information using computerised reminders to the prescriber should be used to improve the use of antiinfectives in the interest of patient safety **(A)**. They ought to be used to reduce consumption and/or costs **(B)**.

Computerised decision support systems that are integrated into the hospital’s internal information system can, by utilising electronic medical records, help to evaluate and optimise the indication for antiinfective therapy, drug selection and dosing **(C)**.

To implement computerised ABS measures, the ABS team must have hospital-wide access rights to electronic medical records (with due respect to data protection).

Significance in practice:

- The local treatment guideline and the antiinfective formulary should be readily electronically accessible from every clinical computer workstation.
- For ABS activities or for surveillance and analysis of antimicrobial usage, computer physician order entry (CPOE) systems should be designed in such a way as to allow automated generation of exact lists of the antiinfectives used.
- Surgical software should be utilisable in such a manner as to ensure that antibiotic prophylaxis is compliant with guidelines.
- Computer-based expert systems cannot replace a physician’s clinical judgement.

## Recommendations of the guideline

### Requirements

#### Availability of a team of ABS experts

**The guideline development group recommends:**

For effective implementation of ABS programmes, it is essential that a multidisciplinary team should be instructed by the hospital administration and allocated with adequate resources to draw up guidelines derived by consensus with the users for the treatment of infectious diseases and to ensure their implementation through ABS strategies **(A)**.

The team should consist of at least one infectious diseases physician (or clinician with infectious diseases training) and an experienced clinical pharmacist/hospital pharmacist, as well as a specialist in microbiology, virology and infection epidemiology being responsible for laboratory diagnostic and microbiological consultation; furthermore, the physician locally responsible for infection control. The team members should either have appropriate training in antibiotic stewardship or already be sufficiently experienced **(A)**.

The team will receive the support and collaboration of the hospital administration, and activities within the ABS programme should be compensated with a minimum of one full-time equivalent (FTE) of 0.5 per 250 beds **(A)**. There should be good collaboration between the Therapeutics and Drugs Committee, Hospital Infection Control Committee, pharmacy and representatives of clinical divisions/departments (ABS representatives), for which purpose the team should issue its own Rules of Procedure **(A)**.

In the community hospital setting, ABS programmes should be available hospital-wide, i.e. involving physicians across all operative and non-operative medical fields. A multidisciplinary team (so-called ABS team) of ABS-trained members (so-called ABS experts) is considered essential to the success of this type of programme. It should have the support of hospital administration, and collaboration of the infection control team and Therapeutics and Drugs committee, the pharmacy and of the responsible physicians (so-called ABS representatives) in the corresponding departments [2, 6]. The advantage of a multidisciplinary team is justified by the necessary diversity of ABS programmes which have different objectives of interventions depending on hospital, type of ward and speciality discipline [26]. At least one randomised controlled [27, 28] and several prospective before-and-after studies [29–35] on the implementation of a trained ABS team, led to a decrease in mortality, a reduction in nosocomial infections and significantly shorter length of hospital stay. In addition, it resulted in an improved quality of prescribing, which in turn, led to fewer drug-related adverse events. The studies show that to achieve different objectives of interventions it is crucial to collect data on clinical, microbiological and prescribing endpoints, and that this can only be done by appropriately trained and sufficiently qualified professionals. Based on the IDSA/SHEA guideline and past experience [6], the ABS team should include at least one infectious diseases physician and a clinical pharmacist, ideally with infectious disease training. The importance of an infectious diseases-trained specialist and clinical pharmacist for effective ABS programmes was shown in several randomised, controlled as well as prospective, quasi-experimental studies. This was demonstrated particularly in regard to appropriate treatment of bacteremia [36], dosage adjustment and early conversion to oral therapy [37–40].

The ABS team should issue Rules of Procedure defining the organisational structures and conditions for implementation of antibiotic stewardship programmes including their functions and objectives. The composition of the multidisciplinary ABS team should be described in detail, from mandate to staffing (qualification, status, objectives and functions, competences and cooperations) and amount of time compensated. Organisational charts can be useful to show internal and external communication structures. The Rules of Procedure should stipulate the frequency of meetings and the reporting obligations toward hospital administration. Potential conflicts of interest of members of the ABS team should be disclosed. Furthermore, it is necessary to lay down hospital-wide rules on how to deal with the pharmaceutical industry or third parties because commercial marketing strategies may possibly influence antimicrobial prescribing [41–43].

Infectious diseases physicians are especially well-suited to planning and implementing ABS programmes and to developing guidelines because of their in-depth knowledge of the treatment of infectious diseases, their broad training in clinical internal or paediatric medicine, and not least their experience in conducting cross-departmental specialist consultations [43]. Infectious disease consultation services improved treatment quality in patients with bacteremia and in some studies also improved survival [36, 45–49]. In the case of community, nosocomial or ventilator-associated pneumonia, introduction of a consultation service in an intensive care unit (incl. training) resulted in shorter length of stay (13.8 vs. 19.2 days), a decrease of ventilation time (7.4 vs. 11.8 days), reduction in duration of therapy (9.2 vs. 14.5 days) and a decrease in mortality by 6–13 % [27, 50] due to optimised empirical and targeted therapy strategies.

Several trails, including a randomised controlled trial, investigated the efficacy of a multidisciplinary ABS team which provides feedback to prescribing physicians. Particularly the feedback from the infectious disease consultation service resulted in a significantly more appropriate antibiogram-based therapy and in discontinuation of antimicrobial therapy [51–54].

Clinical pharmacists/hospital pharmacists are involved in the activities of the Therapeutics and Drugs Committee and in developing local guidelines and formularies. They have special knowledge of pharmacology—such as the clinical relevance of adverse drug effects, dose optimisation or route of administration, and they have experience in conducting audits of antiinfective use, e.g. to ensure guideline adherence [37, 40, 55–57]. Generally, the pharmacist is responsible for design, implementation, and compliance with formulary restrictions and preauthorisation requirements. He is also responsible for processing data on antimicrobial consumption and costs for the purpose of surveillance and benchmarking (pharmacoeconomics) [8, 58–60]. Pharmacist-led parenteral-to-oral conversion programmes resulted in a significant reduction of parenteral therapy duration by 1–1.5 days without negatively impacting clinical outcomes [38, 39, 61]. This can, as shown in surgical departments of a German university hospital, lead to significant cost savings [62–64]. Computerised physician order entry systems (CPOE) could aid the pharmacist in reviewing the appropriateness of antiinfective prescriptions as these systems allow to produce a daily report on the antiinfectives prescribed without review of individual patient charts on the ward.

Ideally, the team is complemented by a medical microbiologist (in Germany: specialist in microbiology, virology and infection epidemiology; in Austria: specialist in infection control and microbiology) and the physician locally responsible for infection control [65]. The expertise of the medical microbiologist is required to establish local guidelines for laboratory diagnostics of infection including preanalytical specimen management, and to report microbiological results in accordance with national and international quality standards. ABS interventions have to be in line with current microbiological diagnostics and reporting, as well as with easily accessible current surveillance data on pathogens. Medical microbiologists should provide support by using targeted diagnostic tests, rapid reporting and professional communication of results. Retrospective investigations indicate that introduction of point-of-care chart reviews focusing on diagnostics delivered by medical microbiologists with infectious diseases training leads to significant reduction in the use of broad-spectrum antibiotics [66, 67].

If infectious diseases specialists are not available in smaller hospitals, an experienced hospitalist can, in collaboration with an authorised pharmacist who has at least 2 years’ working experience in a hospital pharmacy, assume the leadership role in lieu of an infectious diseases physician. In this case, the team members must be ABS-trained, e.g. they must have completed training courses certifying them as ABS experts with knowledge in the following areas: design and implementation of ABS tools (treatment guidelines, antiinfective formulary, treatment pathways), application and implementation of point prevalence surveys of antiinfective prescribing practices, requirements for surveillance data (consumption, pathogens, resistance), the content of current guidelines and important ABS intervention strategies. The ABS experts should be capable, as a team, of developing and implementing a programme for continuous improvement of the quality of antiinfective prescribing that is tailored to the specific needs and situation of the respective hospital. Relevant continuous training in the field of ABS is recommended.

Based on the available evidence from the literature and repeated internal consultations, the guideline development group recommends that clinical infectious diseases physicians or clinical pharmacists with ABS training should principally assume core leadership function in the ABS team. Transferability of available experience (mainly American) to the German health care system is limited. The German Society for Hygiene and Microbiology (DGHM) and the Paul Ehrlich Society for Chemotherapy (PEG) point out that the conditions in Germany (several years further training in medical microbiology, medical microbiologists working in hospital and also providing infectious disease consultation, likewise the shortage of infectious diseases physicians and specialised pharmacists) and experience in some other European countries (e.g. England and the Netherlands) should allow to consider clinically oriented and experienced medical microbiologists (German: specialist in microbiology, virology and infection epidemiology), as being suitable for core leadership function, assumed they are for the most part present and available in the hospital and released from duties in the laboratory.

The team size depends primarily on the size of hospital. In the older and the current literature, between 0.5 and 1.5 full-time equivalent posts depending on the number of beds (~200 to ~900) or level of care provided, equating to one full-time equivalent of 0.5 per 250–300 beds, is well documented as being cost effective and associated with high net savings in the initial phase [23, 27, 29, 68–71]. Ongoing activity by the teams is essential to preserve the effects, as the quality of use and cost usually deteriorate rapidly when ABS programmes are discontinued [23, 72]. In larger hospitals, it is therefore recommended to appoint department-specific ABS representatives to support the ABS team in its activities. The ABS team must be involved in decision-making in the Therapeutics and Drugs Committee and the Hospital Infection Control Committee as these committees may influence the design of ABS strategies, and jointly coordinated programmes (involving bundles of interventions) must be discussed to be effective particularly in the area of nosocomial infections. In the event of *C.difficile* outbreaks, or if the *C. difficile* incidence increase over time, infection control strategies alone are often not sufficiently effective. As demonstrated in multiple time-series analyses, restricting use of cephalosporins, fluoroquinolones or clindamycin is necessary to reduce *C.difficile* incidence effectively (see 2.1., 3.3.) [2, 25, 73].

The recommendations of the former ABS Group Austria on the further development of ABS programmes in Austrian Hospitals (“Antibiotika-Kultur in Krankenanstalten”) [74], the IDSA/SHEA Guideline on “Hospital Antibiotic Stewardship” [6] and the Australian recommendations [43] refer to the need for hospital administration to direct the ABS team to plan ABS activities, to support the implementation of these interventions and to provide necessary resources. Several quasi-experimental before-and-after studies emphasise the importance of support given by the hospital administration or departmental management, in particular in the development of guidelines, their establishment and successful implementation [75, 76].

#### Availability of surveillance data on pathogens, resistance, and antimicrobial consumption

##### Pathogens and resistance

**The guideline development group recommends:**

Antimicrobial susceptibility data on major pathogens should be available and accessible at least yearly on a hospital-wide level and separately for general and intensive care units, or department-specific, as the case may be. Data on primary isolates should be shown by pathogen and type of specimen, e.g. blood, urine, miscellaneous samples. Culture results from screening tests should be shown separately. Susceptibility rates should indicate the number of isolates tested. Infection rates should relate consistently to a single denominator (e.g. patient-days/number of cases). Participation in an established surveillance system is recommended **(A)**.

A requirement for successful ABS programmes is the availability of current hospital-wide data on pathogens and antiinfective use. This will allow for weak-point analysis and optimisation potential [2, 8, 77]. In addition to provision of routine reporting on pathogen identification with antibiogram, the microbiology laboratory is, in coordination with the ABS team, responsible for surveillance of pathogen and resistance patterns. Expert consensus recommends that pathogen-specific susceptibility data should be updated at least annually. Data on primary isolates and subsequent isolates should be presented separately. In addition, data should include susceptibility and resistance rates according to generally recommended breakpoints as well as the number of isolates tested. Electronic data processing available in the microbiology laboratory can facilitate unit-specific (general ward vs ICU) or department-specific evaluation of resistance. This allows to recognise the distribution of individual pathogens and antibiotic susceptibility profiles in different departments in dependence on prescribing habits, which helps guide ABS interventions.

The available infrastructure and personnel resources must allow even hospitals without an on-site microbiology laboratory, to provide hospital-based or unit-based data on pathogens and antimicrobial susceptibility, if need be, at shorter intervals, whereby presentation of data on susceptibility rates on fewer than 10 tested isolates does not appear useful.

Expert consensus recommends reporting at least on *S. aureus*, *E. coli* other *Enterobacteriaceae*, *P. aeruginosa* and *Candida* spp. by specimen type (blood, urine and miscellaneous samples) as well as on *C. difficile*, whereby screening culture results should be reported separately. Standardised surveillance is a fundamental requirement for benchmarking with other institutions/departments. Interpretation of the data takes into account the size of hospital, the level of care, and the patient mix (e.g. hematologic-oncologic patients). Participation in established surveillance systems is recommended.

##### Antimicrobial consumption

**The guideline development group recommends:**

Data on antimicrobial consumption, expressed as use density (daily doses per 100 patient-days) should be collected at least annually or preferably quarterly and are generally reported by the pharmacist. Data are reported institution-wide, at the ward level as well as for individual (speciality) departments. On demand, data should be broken down to the agent level and should be provided to the ABS team. Participation in an established surveillance system is recommended **(A)**.

Point prevalence surveys should be conducted for systematic quantitative and qualitative assessment of antiinfective use, and, if required should be reevaluated short-term **(A)**. Antiinfective use data are collected at the patient level, allowing to assess prescribing quality based on indication and type of infection, and to recognise the need for targeted ABS strategies. Access to patient-level data ought to be guaranteed.

Continuous reporting of surveillance data on antiinfective consumption is useful in monitoring trends and identifying areas for evaluating appropriateness of prescribing. It therefore supports systematic audit of antimicrobial use with intervention and feedback to the prescriber [78–80]. Consumption data are usually obtained from the pharmacy and are being presented as daily doses by the pharmacist. They are an essential prerequisite for medium and long-term assessment of the effectiveness of interventions [81]. Another goal of continuous surveillance is early identification of an increase in antibiotic consumption.

**Fig. 1:**
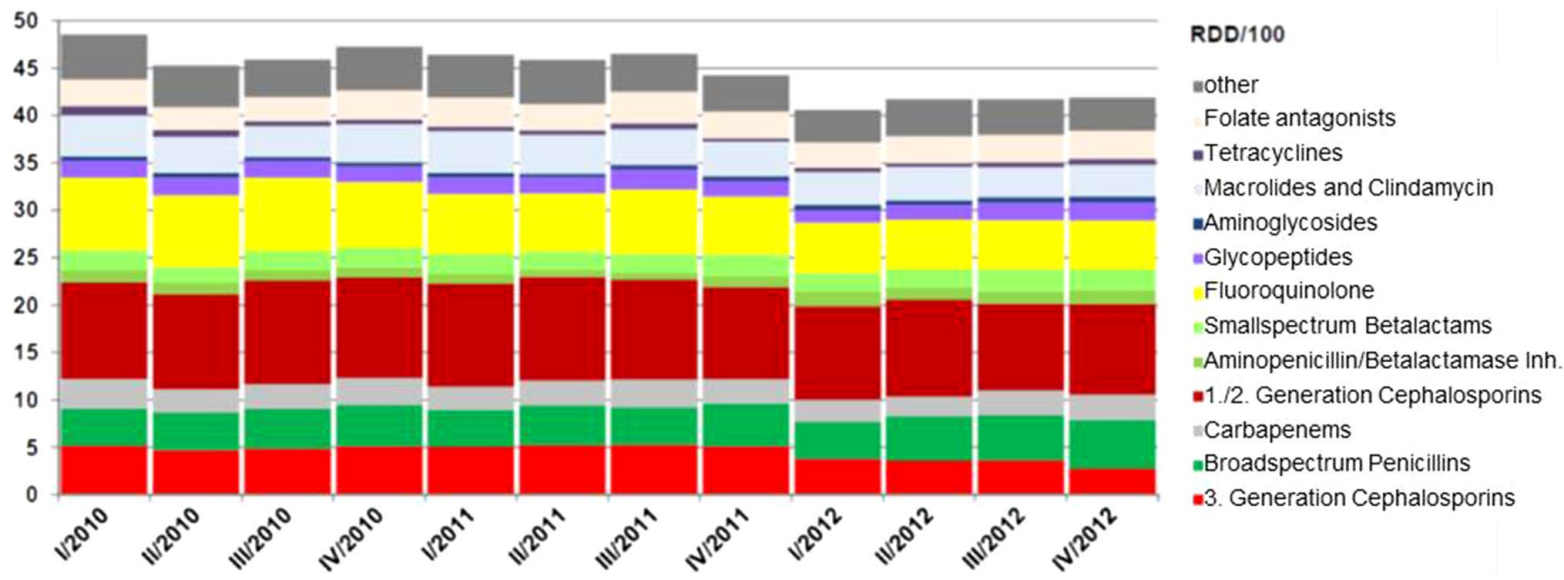
Graphical presentation of quarterly use density (RDD/100 patient-days) for different antibiotic classes

Expert consensus recommends that these data should be available institution-wide, for individual departments and at the ward level (e.g. general ward, intensive care unit) at least annually, preferably quarterly. Data should be collected by antimicrobial agents and reported in the form of daily doses per 100 patient-days (e.g. defined daily doses, DDD, according to the ATC Index of the WHO, and/or recommended daily doses, RDD) [82]. Upon the request of the ABS team, aggregate antibiotic usage data should be available for specific classes of antibiotics as well as stratified by different clinical units. Good examples for this form of data presentation such as so-called “antiinfective report” incl. graphical presentation are available for various German hospitals (Fig. 1).

Economic data (e.g. antibiotic costs) ought to be also documented; however, these data alone do not provide a suitable basis for analysis and intervention in terms of ABS. According to Article 23 (4) of the Infection Protection Act, usage data must be evaluated taking into account local resistance data and appropriate conclusions must be drawn regarding the use of antibiotics. Furthermore, the necessary adjustments of antibiotic consumption must be implemented and the staff must be informed. Participation in an established surveillance system provides a standardised method to calculate antimicrobial use density and is therefore recommended. Thus, depending on the patient mix, comparisons between different hospitals are also possible [81, 83]. However, IT-based patient-level consumption data, so-called prescribed daily doses (PDD) should be the ultimate goal.

Point prevalence surveys can be very helpful for temporary assessment of the quality of antimicrobial prescribing, e.g. before and after guideline amendment [84–86]. A point prevalence survey provides information on the choice of substance, dose, dosing interval and route of administration. In addition, data on the indication for prescribing (nosocomial vs community acquired or prophylactic) and the type of infection can be collected at patient level, allowing to evaluate the consumption density in relation to the prescribing quality. The ABS team ought to have access to relevant patient data to conduct these surveys which are usually carried out as a 1-day point prevalence survey. Starting from the day of the survey, prescription data can also be collected retrospectively for a limited time interval (e.g. 6 days). On the day of the survey, patient-based data as mentioned above are documented. Additionally, it is recommended to document the number of patients per unit, to calculate the prevalence of antiinfective prescriptions per unit (e.g. ICU, department). This analysis allows to evaluate the relation of defined daily doses recommended by the WHO (DDD) to prescribed daily doses (PDD) derived from chart review. Additionally, other patient-relevant information can be investigated, e.g. on immunosuppression, organ insufficiencies or on presence of devices. European 1-day point prevalence surveys (http://www.esac.be, http://www.ecdc.europa.eu/en/healthtopics/Healthcare-associated_infections/database/Pages/database.aspx) have shown that approximately one-third of all hospitalised patients received antiinfective treatment and that even on regular wards >50 % of total consumption was given intravenously. Furthermore, within hospitals fluoroquinolones and cephalosporins were prescribed frequently, more than 30 % of the patients received combination therapy, and >50 % of perioperative prophylaxis was administered longer than 1 day. Only 62 % of patients were treated in adherence with guidelines [85–87]. Point prevalence surveys can also be used to verify the feasibility of quality indicators (see Sect. 2.2.4).

### ABS core strategies

Most previously published experience with ABS programmes in hospital shows that sustained efficacy can be achieved under the requirements mentioned above and on the basis of generally accepted strategies or bundles of strategies. Certain components of ABS programmes are considered and prioritised as core ABS strategies, while others are considered optional or supplemental [2, 6, 22, 25]. The following core strategies are recommended by the guideline development group.

#### Application of local treatment guidelines/pathways, hospital antiinfective formulary, formulary restriction and approval requirements

**The guideline development group recommends:**

Developing and updating local treatment guidelines, clinical pathways, and an antiinfective formulary is one of the ABS team’s chief responsibilities. The antiinfective formulary should be based on national and international guidelines as well as on the local/regional pathogen and resistance patterns, and possibly drug costs. Drugs on the antiinfective formulary should be categorised according to recommended versus reserve or special compounds. In addition, these should be tagged with special prescription status and be subject to approval and preauthorisation requirements. The antiinfective formulary is updated at least yearly based on therapy guidelines and whenever necessary and approved by the Therapeutics and Drugs Committee **(A)**.

Adherence to guidelines regarding substance selection, dosing, route and duration of treatment may improve clinical outcome in terms of mortality, as well as treatment duration and length of hospital stay. To ensure adherence, users should be involved in developing the guidelines and be educated through audits of antiinfective use or antiinfective point-of-care chart reviews **(A)**.

Individualising antiinfective prescriptions with or without special approval requirements improves targeted therapy and reduces inappropriate treatment. Various possibilities for implementation have been described and should be used, from simple antimicrobial order forms to highly differentiated antiinfective request forms that may be subject to specific time limits or limited to certain hospital areas **(A)**. Guideline-based antiinfective drug use or use of individual defined substances can be controlled by this means, thus minimising consumption, costs and adverse drug events.

Restricting whole substance classes can—by shifting to an alternative substance—prove to be an effective strategy for controlling nosocomial infections and the development of critical resistance levels; accordingly, antiinfective restriction ought to be targeted **(B)**. At the same time, routine surveillance of antibiotic consumption and locally prevalent pathogens and their susceptibility patterns should be performed to detect possible adverse effects of the strategy in time **(A)**.

Local treatment guidelines and clinical pathways are established and regularly updated by the ABS team with the involvement of the ABS representatives delegated from other clinical departments. National and international guidelines, the patient mix and local microbiology and resistance patterns should be taken into account. The established or revised local guidelines should have institution-wide validity for which consensus must be obtained. The treatment guidelines are presented to the Therapeutics and Drugs Committee and the Hospital Infection Control Committee. It is recommended to provide local treatment guidelines in electronic or pocketbook format and to ensure acceptance among users through training and education [88]. Without these measures guideline adherence is rather poor, and effects in terms of improving clinical outcomes or other endpoints remain small [89].

Treatment guidelines or clinical pathways can improve outcomes related to mortality, length of hospital stay and duration of treatment [90, 91]. High adherence to guidelines or clinical pathways, e.g. for management of community-acquired or nosocomial pneumonia, can be achieved with training and education. Thus, mortality can be decreased and the medium duration of therapy and hospital stay can be shortened by 1.7–6.8 days, while antiinfective usage is reduced by up to 77 % [92–98]. Various strategies of treatment optimisation have been studied for community-acquired or nosocomial pneumonia [99–102] and have partly been addressed in international and national guidelines. Their implementation in local guidelines, guideline adherence assumed, can help to avoid that therapy is either too broad or too long. An American and a French observational study have shown that involving physicians in the development of local guidelines can improve acceptance. When local consensus guidelines were posted on the intranet and regularly distributed to physicians and presented in departmental staff meetings, guideline-conform management of nosocomial pneumonia increased from 46 to 81 %, and 14-day mortality dropped from 23 to 8 % [103]. In a study of endocarditis, compliance with antimicrobial therapy improved from 23 to 62 % and 1-year mortality significantly decreased from 19 to 8 % [104] (Table 1). Numerous new investigations on improving guideline compliance have shown that institutionalising guidelines can optimise the quality of therapy in different categories (e.g. dose adjustment to renal function, parenteral-to-oral conversion, timely administration) by about 10 or more percent [105–110].

**Table 1:** Examples for use of treatment guidelines and clinical pathways

References	Study-type/evidence	Patients	Intervention	Endpoints	Results
Soo Hoo et al. [103]	Observational study (II)	Patients with community-acquired pneumonia (58 patients before intervention, 58 patients after intervention)	Establishment of guidelines for the diagnosis and management of nosocomial pneumonia	Mortality Proportion of patients with guideline-conforming treatment	Lower mortality rate at 14 days (23 vs 8 %, *p* = 0.03) Increase in the number of patients treated in conformity with guidelines (46 vs. 81 %, *p* < 0.01)
Botelho-Nevers et al. [104]	Observational study (II)	Patients with infectious endocarditis (173 patients before intervention, 160 patients after intervention)	Establishment of treatment guidelines for management of infectious endocarditis	Mortality Guideline adherence (compound selection, duration of treatment)	Lower 1 year mortality (18.5–8.2 %, HR 0.41; 95 % CI, 0.21–0.79, *p* = 0.008) Lower hospital mortality (12.7–4.4 %, *p* = 0.007) Increase in guideline adherence: compound selection (31.6 % auf 95 %, *p* < 0.001) Compound selection and duration of treatment (22.7 % auf 61.8 %, *p* < 0.001)
Marrie et al. [93]	Randomised, controlled study (i)	Patients with community-acquired pneumonia in the emergency room of a hospital (nine hospitals with clinical pathway, 10 hospitals without clinical pathway)	Establishment of a clinical pathway for treatment of community-acquired pneumonia in the emergency room of nine hospitals	Mortality Length of hospital stay Duration of treatment Proportion of patients with monotherapy	No difference in mortality Shorter length of hospital stay by 1.7 days (6.1–4.4 days, *p* = 0.04) Shorter duration of treatment by 1.7 days (6.3–4.6 days, *p* = 0.01) Increase in the proportion of patients with monotherapy (27–64 %, *p* < 0.001)
Singh et al. [102]	Randomised, controlled study (I)	Patients with ventilator-associated pneumonia (39 patients treated in accordance with a risk score-based clinical pathway, 42 patients received standard therapy)	Establishment of risk score-based clinical pathway	Mortality Length of hospital stay (ICU) Detection of MDR pathogens Duration of treatment, costs	No difference in mortality Shorter length of hospital stay (ICU) by 5.3 days (14.7–9.4 days; *p* = 0.04) Reduced detection of MDR pathogens (38–14 %, *p* = 0.017 Shorter duration of treatment (9.8–3 days, *p* = 0.0001) Lower treatment costs (640$–259$, *p* = 0.0001)
Ibrahim et al. [97]	Observational study (II)	Patients with ventilator-associated pneumonia (50 patients before intervention, 52 patients after intervention)	Establishment of a treatment guideline for management of ventilator-associated pneumonia	Mortality Length of hospital stay, antibiotic therapy complying with guidelines, Duration of treatment	No difference in mortality Increase in the proportion of antimicrobial therapy conforming to guidelines from 48 to 94.2 % (*p* < 0.001) Shorter duration of treatment from 14.8 days ± 8.1 days to 8.6 ± 5.1 (*p* < 0.001)

Clinical pathways complement local treatment guidelines, often taking into account diagnostic algorithms and risk scores. They are designed as a flowchart to simplify and improve the management of patients with infectious diseases. In a controlled, multi-centre Canadian study a risk score (PSI, pneumonia severity index)-based clinical pathway was instituted, addressing criteria for inpatient admission, sequential therapy and discharge of patients with community-acquired pneumonia. Although patients in the “experimental” arm had more severe disease, hospital stay and duration of parenteral antibiotic therapy was significantly shortened in this patient group, and the patients received monotherapy significantly more often without negative impact on mortality. Within this framework, an Australian study showed an approximately 10 % reduction in the use of broad-spectrum antibiotics [93, 111, 112]. Similar results were achieved by a more recent observational study in the UK, where introduction of a risk score (CURB-65)-based clinical pathway for treatment of community-acquired pneumonia influenced prescribing behaviour. As expected, CURB65-guided therapy resulted in an overall reduction in the prescription of cephalosporins and macrolides by 19 and 14 %, respectively, without negatively affecting outcome (30-day mortality, clinical response, treatment outcome). There was a corresponding increase in use of aminopenicillin monotherapy, and guideline compliance increased from 25 % to over 60 % [113].

Acceptance and implementation of treatment guidelines not only improves by involving users in guideline development. Other supplemental ABS strategies such as repetitive education, training and audits of antibiotic prescribing with feedback to the prescriber improve acceptance and adherence [114]. This is shown by a controlled before-and-after study in which adherence was consistently improved by a combination of interventions involving distribution of information packs to staff, repeated compilation of prescription data and educational sessions followed by reminders in the form of posters [98]. Implementation of a uniform guideline for perioperative prophylaxis including recommendations for choice of agent, dosage and timing resulted in annual antimicrobial cost savings of approximately USD 112,000 in a 1400-bed hospital [115].

The institutional antiinfective formulary is established by the pharmacist in the ABS team based on therapeutic efficacy, toxicity and cost. Drugs of the formulary should be categorised into recommended versus reserve or special compounds depending on local treatment guidelines. Graphical overview with alerts (traffic light system), information on daily therapeutic costs or restrictions on use is advisable. Adding information on special prescription or approval requirements is desirable. Besides information on agent and trade names, these lists contain information on the recommended daily dose, including dose adjustments in regard to organ impairment (Table 2). The antiinfective formulary must be passed by the Therapeutics and Drugs Committee. The formulary has an immediate influence on prescribing behaviour [116].

**Table 2:**
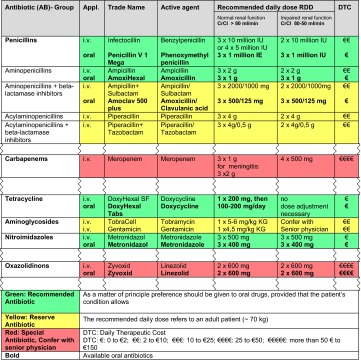
Example of a formulary

Caution should be exercised in controlling antibiotic use via the formulary alone without an indication-based treatment concept and concomitant surveillance of antibiotic consumption and resistance. It was for instance observed that by adding levofloxacin to the antiinfective formulary fluoroquinolone use subsequently increased substantially, resulting in a higher rate of MRSA infection. When an alert was inserted next to the fluoroquinolone selections on the electronic order entry screen, indicating alternative antibiotic agents in accordance with local guidelines, levofloxacin use decreased again by 50 % from 12 to 6 DDD/100 patient-days and the MRSA infection rate decreased again from 1.37 to 0.63 cases per 1000 patient-days [117]. Similar effects have been observed for other substances and classes and pathogens [118, 119].

Individualised antiinfective orders with or without approval requirements extend from simple to highly differentiated, computer-assisted order forms with an automatic prescription stop after a defined time (so-called “automatic stop order”). These can be agent, patient or indication based, temporary or limited to certain hospital areas. Individualised antiinfective orders present an effective tool to quickly and effectively influence prescribing behaviour. Special order forms or approval requirements are usually implemented for broad-spectrum antibiotics, new/expensive substances or substances requiring extensive consultation. They require justification for prescription, which must be evaluated prior to approval, and can effectively control use and costs. These substances are separately marked in the antiinfective formulary.

Many older prospective before-and-after trials dating from the 1980s and 1990s documented that restricting use of new and expensive cephalosporins generated cost savings of between 19 and 46 %, and reduced consumption by up to 50 % [6, 120–124]. Significant cost reductions being achieved through an antimicrobial-restriction policy are less commonly observed in recent years, because numerous antibiotics have lost patent protection. Nevertheless, more recent studies showed continuing effectiveness regarding reduction in antibiotic consumption of as much as 54 %. [23, 125–127]. Newer research on restricting use of broad-spectrum antibiotics yielded a monthly reduction from 137 to 72 DDD/100 cases or from 181 to 102 DDD/1000 patient-days, respectively. Overall, after implementation, the ABS programme delivered effective cost savings of USD 300,000 p.a. (corresponding to net savings of USD 2350/100 cases or 2182/1000 patient-days, respectively). [59].

Use of special order forms limiting antibiotic duration has proved to be particularly effective within the field of perioperative antibiotic prophylaxis. In several prospective, quasi-experimental before-and-after studies the effect of automatic stop order forms on antibiotic consumption, costs and guideline adherence to avoid extended prophylaxis was evaluated. By educational training, an overall 20–30 % improvement in guideline adherence was observed with respect to choice of drug and duration of antimicrobial use, with one study also showing improvement in appropriate timing of perioperative antibiotic prophylaxis before incision. This resulted in a reduction of surgical site infections from 3.2 to 1.9 %, a reduction in cost of USD 3000/100 patient-days and a reduction in consumption of approximately 20 DDD/100 patient-days [128–132]. Other equally effective automated stop orders limiting total duration of treatment (e.g. 14 days) or restricting duration of reserve drugs such as vancomycin or carbapenems (72 h for empiric therapy, 7 days for therapeutic indication) have been described. Treatment beyond was only possible following consultation with the infectious diseases specialist or pharmacist. As a result, consumption of these substances was reduced by 10–25 % [125, 133, 134].

Specific programmes to restrict antimicrobial use can minimise nosocomial infections (e.g. *C.difficile*) and the increase of resistant pathogens (ESBL, MRSA) by a rapid and marked alteration in consumption. However, such programmes are usually only temporary and lack sustainable efficacy [22]. Restriction strategies are adopted in coordination with the Therapeutics and Drugs Committee, Hospital Infection Control Committee, the pharmacy, and hospital management. Timely and continuous surveillance of consumption, infectious diseases and resistance data are to be assured, to monitor compliance, but also to be able to rapidly identify possible negative impacts. The importance of instituting a programme for the surveillance of antimicrobial use including unrestricted antibiotics, cost and the development of resistance demonstrated by a prospective quasi-experimental observational study at a 450-bed hospital in Greece. In the study, use of carbapenems, third-generation cephalosporins, and fluoroquinolones was restricted based on a national recommendation in context of growing resistance among Gram-negative microorganisms. As a result, ciprofloxacin and ceftazidime consumption decreased as desired by 28 and 42 %, respectively. Subsequently, susceptibility of *P. aeruginosa* (32–45 %) and *E. coli* (77–84 %) to ciprofloxacin increased. On the other hand, susceptibility of *K. pneumoniae* to ciprofloxacin (80–60 %) and ceftazidime (61–46 %) continued to decrease. Of note, piperacillin/tazobactam use increased by 271 % and overall costs and consumption were 12–13 % higher than before intervention [135, 136].

Programmes restricting use of cephalosporins and fluoroquinolones have been repeatedly examined for their “ecological” effects [137–140]. Multicenter controlled investigations in France show a 90 % reduction in fluoroquinolone use after introduction of a time-limited restriction, resulting in a significant reduction in MRSA. Reintroduction of fluoroquinolones was associated with a significant increase in MRSA compared to the previous period [141, 142]. A new study from France shows that even less restrictive fluoroquinolone use (20 % reduction) combined with improved hand hygiene also reduces the rate of MRSA (moderate) and at the same time impacts positively on resistance of *P. aeruginosa* to fluoroquinolones [143]. Other new studies demonstrate effects of changes in fluoroquinolone prescribing practice on *C. difficile*-associated diarrhoea [144–148]. The effects, however, are not always due to the fluoroquinolone reduction alone.

#### Design and implementation of education, training and information

**The guideline development group recommends:**

Targeted education, training and information are essential elements of any ABS programme. They provide the foundation of knowledge needed to promote more rational use of antibiotics and reasonable microbiological diagnostic, and to improve acceptance of ABS programmes. They have the objective of optimising the therapeutic and diagnostic management of patients with infection through greater adherence to recommendations. They should preferably take place as an active training measure rather than in the form of passive communication of information **(A)**.

Education, training and information in different formats and on various topics should be offered repeatedly as they are not sustainable as a one-off measure. They should be organised in agreement and integration with local ABS programmes **(A)**.

Education, training and information should be independent of commercial interests, whereby the hospital administration is responsible for implementing and financing the measures **(A)**.

Education, training and information are essential elements of every ABS programme. Overall, in a systematic review active clinician education in the form of lectures, seminars, “bedside teaching” demonstrated greater effectiveness than passive education techniques like posters, pocket cards or written prescription recommendations [149]. Two multicenter, randomised, controlled, and some before-and-after studies demonstrated that an educational intervention improved compliance with guideline-recommended diagnostic, therapeutic and prophylactic measures and resulted in a reduction in the number of non-indicated treatments [150–152]. By educational training of nurses and medical staff inappropriate submission of urine cultures decreased from 2.6 to 0.9 per 1000 patient-days; treatment of asymptomatic bacteriuria was reduced from 1.7 to 0.6 per 1000 patient-days, while in another study a significant reduction from 74 to 17 % was seen [153, 154]. In Canadian long-term health care facilities, a 1-year long educational intervention involving repeated mailing of antibiotic guidelines with feedback on individual antibiotic prescribing behaviour of urinary tract infection, pneumonia, skin and soft tissue infection and sepsis resulted in a significant 64 % reduction of nonadherent treatment compared to control facilities [155]. Nonadherent antibiotic prescriptions remained lower during follow-up, although after termination of the educational intervention, the effect was no longer significant compared to control facilities. A similar effect was achieved in a study on an educational programme for guideline-based treatment of respiratory tract infections in emergency departments, in which 1 year post-intervention a 10 %, albeit non-significant reduction in antibiotic consumption was still documented compared to sites without intervention [156].

With the aim of reducing extended use of perioperative antibiotic prophylaxis by means of information disseminated by e-mail, poster and lectures, 12 Australian hospitals succeeded in rapidly and effectively limiting the duration of antibiotic prophylaxis to maximally 48 h, thus achieving considerably lower costs, which more than outweighed the costs of the 1-year intervention [157]. However, the effect of the intervention rapidly declined with time, as seen in other studies [158]. Repeated guideline-based educational interventions are necessary. They were shown to be particularly effective in optimising perioperative antibiotic prophylaxis [6, 159]. In an Argentine multi-step ABS programme involving training and formulary restriction, antimicrobial consumption could be reduced from 43 to 28 DDD/100 patient-days, resulting in substantial savings (>900.000 USD) over 18 months. During the training period a significant increase in the rate of prescriptions based upon microbiology results (27–63 %) was found, and use of ceftriaxone and carbapenems subsequently more than halved [30]. The combination of one-on-one education (academic detailing) and special review of orders for either levofloxacin or ceftazidime was also seen as a highly effective method for reducing inadequate antibiotic use. Unnecessary antibiotic prescription was significantly reduced by 41 % with no change in clinical outcome [28, 160]. However, academic detailing is time-consuming and personnel-intensive [28, 160]. This can partly be compensated by less time-consuming feedback activities, e.g. in the form of written recommendations placed in the patient chart; however, these are not quite as effective and less sustainable [157].

Education, training and information should be independent and should not be guided by the commercial interests of the manufacturers of medical and diagnostics products, since this is the only way to ensure that prescribing and professional behaviour are not subject to direct or indirect influence (Table 3). A systematic review examined the impact of various strategies undertaken by the pharmaceutical industry such as visits, funding for travel or lodging, sponsoring educational events, free samples, etc., on prescribing practices. According to the study, industry-sponsored continuing medical education (CME) had the biggest impact on physician prescribing practices compared to other activities, leading to a 6–19 % increase in prescription rates of the sponsor’s medication [41, 42]. The responsibility of organising, holding and financing educational events should be assumed by hospital management.

**Table 3:** Examples of the influence of commercial interests on prescribing and formulary design [41, 42]

Meetings with pharmaceutical representatives	66 % less likelihood of prescribing generic products
Travel sponsoring (congresses, etc.)	Requests to add the sponsor’s drugs to the hospital formulary are associated with an odds ratio of 7.9 % (95 % CI, 1.1–55.6) 4.5- to 10-fold increase in hospital prescribing rate (sponsor’s products) as compared to before travel
Continuing medical education (CME funding)	Prescribing rate, (sponsor’s products) increases by 5.5–18.7 %
Research funding	Requests to add the sponsor’s drugs to the hospital formulary is associated with an odds ratio of 9.5 (95 % CI, 2.6–35.7)

#### Conducting proactive audits of antiinfective use

**The guideline development group recommends:**

Proactive on-site audits of antiinfective use in the context of antiinfective point-of-care chart reviews are important elements of ABS programmes and should be performed routinely by the ABS team **(A)**. They enhance compliance with guidelines or clinical pathways, improve outcome in patients with infection and improve the quality of prescribing with regard to indication, choice of agent, dosing, dosing interval, administration route and treatment duration.

Depending on the problem and treatment target, besides point prevalence studies, agent-, indication- and/or diagnosis-related audits of antiinfective use should be conducted within the scope of regular antiinfective point-of-care chart reviews either hospital-wide or at the unit level, whereby quality indicators should preferably be applied **(A)**.

Results should be fed back in direct interaction with prescribing physicians and discussed with them **(A)**.

Proactive audit of antiinfective use with review and feedback include the collection and analysis of data on diagnosis, indication, choice of agent, dosing, administration route and treatment duration at patient level. The results are fed back to and discussed with the prescribing physicians (point-of-care interventions). In personal consultation with the prescribing physicians reasons for choice of drug could be asked for and therapy should be optimised based on clinical, laboratory, radiological and microbiological examination results. Concomitant disease, comedication, expected pathogens when microbiology is not yet known and local antimicrobial susceptibility patterns must be taken into account. The guideline of two North American medical societies has described this type of audit as a highly effective interventional tool that provides a core strategy for an antimicrobial stewardship programme—called “prospective audit with intervention and feedback” [6].

Proactive audit of antiinfective use with review and feedback by an ABS team has been described as effectively increasing the rate of adequate antiinfective use by 20 %; the strategy can reduce the rate of inadequate use by half [34, 53, 161–164]. Methods of feedback can be modified, especially when computer-based assistance is available [165]. The quality of information is important; however, personal feedback is often more effective [165, 166]. In addition to a direct improvement in the quality of prescribing, audits of antiinfective use allow to recognise the need for education and training.

Audits of antiinfective use can be agent-, diagnosis- or indication-based and can be performed at the patient level, in individual departments, wards, or hospital-wide. Targeted (e.g. in relation to agent, speciality department or ward) as well as time-restricted antiinfective audits can be highly effective (examples are shown in Table 4). A programme in which an infectious diseases specialist or pharmacist conducted targeted point-of-care chart reviews (3×/week) of patients receiving multiple antibiotics, prolonged or high-cost therapy (120-bed hospital), achieved good acceptance: 69 % of the recommendations were accepted and implemented, of these 38 % were to discontinue therapy due to excessive duration, duplicate coverage or inappropriate use, and 33 % were to switch to oral application. Compared with the previous year, a cost reduction of 19 %, estimated savings of USD177,000 were achieved [167].

**Table 4:** Examples for performing targeted proactive audits of antiinfective use

• Perioperative antibiotic prophylaxis in selected surgical fields	
• Targeted therapy of bacteremic patients hospital-wide	
• Community-acquired pneumonia in the emergency department	
• Sequential therapy on general wards with antibiotics of high bioavailability	

Agent-related proactive audit of antiinfective use can address dose adjustment to organ dysfunction, switch to oral application, discontinuation of therapy or targeted therapy based on microbiology. In a multicenter, randomised, controlled study parenteral antibiotics could be significantly reduced by 1 day when patients who had received parenteral antibiotics for longer than 3 days were reviewed by an infectious diseases physician for possible sequential therapy based on defined clinical and laboratory criteria and a recommendation was made for switch to oral drug application [38]. The intervention showed lack of effect on length of hospital stay, but reduced mean antibiotic costs per patient significantly from USD 36 to USD 20. Following updated recommendations on aminoglycoside treatment, antiinfective visitations by infectious diseases physicians achieved a significant 11 % reduction in nephrotoxicity by shortening the treatment duration from 6 to 4 days and optimise dosing by monitoring drug levels [161]. In a study assessing the effects of intervention and feedback by the infectious diseases physician, empiric treatment with levofloxacin, vancomycin and carbapenems was switched to targeted antibiotic treatment in line with guidelines. Consumption subsequently decreased by 20 % and median duration of therapy was reduced from 6 to 4 days in comparison to a control group [168, 169]. In another intervention, a prospective audit and feedback programme was instituted in a teaching hospital by pharmacists and infectious diseases physicians to counteract a trend towards increasing use of expanded-spectrum antimicrobials. This resulted in a significant reduction in consumption of third-generation cephalosporins and aztreonam within a period of 6 years from 28 to 6 DDD/1000 patient-days. Furthermore, there was a significant decrease over time in infections caused by *C. difficile* from 2.2 to 1.4 cases/1000 patient-days [29]. According to another recent time-series analysis, a significant decrease in fluoroquinolone consumption from 118 to 78 DDD/1000 patient-days over 4 years was achieved by the ABS team following implementation of daily hospital-wide audits of fluoroquinolone use based on individual patient data. At the same time, the rate of fluoroquinolone-resistant *P. aeruginosa* continuously decreased from 42 to 26 % [143]. A similar intervention in intensive care units resulted in a sustained 22 % decrease in the number of days of therapy with extended spectrum antibiotics compared with the control group—without negative impact on mortality [170] (Table 5).

**Table 5:** Suggested evaluation categories in local audits of antimicrobial use

1.	Antimicrobial therapy adheres to established institutional guidelines with respect to:
	Choice of agent
	Dose
	Route of administration
	Duration of infusion
	Duration of treatment
2.	Antimicrobial prophylaxis adheres to established institutional guidelines with respect to:
	Choice of agent
	Dosing
	Route of administration
	Timing of preoperative dose
	Dosing interval
	Duration of administration

With proactive audit of antimicrobial use focussing on diagnosis and indication for antibiotic treatment by an infectious diseases physician-led ABS team with direct interaction and feedback as well as written documentation of recommendations, length of stay was shortened by 3.3 days and a 6 % decrease in mortality was achieved [27]. As a result of the intervention, median hospital costs were reduced by USD 2642/intervention. By optimising the process of perioperative antibiotic prophylaxis, appropriate dosing and timely administration significantly increased from 72 to 90 % and 36 to 79 %, respectively [171]. Within the frame of quality assurance, and for benchmarking purposes with other hospitals, targeted audits of selected process of care indicators for the management of important and frequent infections can also in small acute-care hospitals lead to a significant improvement in adherence to established guidelines. In a quasi-experimental before-and-after study of a total of 36 hospitals (<200 beds) the effect of proactive audit and feedback, by using quality indicators, on the management of pneumonia in the emergency department was investigated. The hospitals demonstrated a 30 % improvement in the performance of microbiological diagnostics (blood/sputum cultures) prior to therapy and antibiotic administration within 4 h of hospital admission. As a consequence, mortality was reduced significantly by 12–6 % [172]. Proactive audits of antiinfective use based on selected quality indicators should regularly take place (see Sect. 2.2.4).

The ABS team should determine the objective, type, contents and frequency of point-of-care chart reviews in agreement with the wards or departments involved and should give report on its effects. The ABS team should get project-specific to hospital-wide access to the laboratory, radiological and microbiological data needed. Computer-based information technology can facilitate audits of antiinfective use (see Sect. 3.3.4)

#### Quality indicators

**The guideline development group recommends:**

ABS programmes should be integrated within the hospital’s quality management. Content overlaps with the Therapeutics and Drugs Committee (drug safety) and Hospital Infection Control Committee (prevention of nosocomial infection) is useful and desired. Appropriate quality indicators to measure prescription practice (process measure), emergence of resistance or trend in consumption (outcome measure) and structure ought to be set and applied in every ABS programme **(B)**. At least three indicators measuring structural quality and at least three indicators measuring process quality should be set regularly **(A)**.

ABS programmes are to be regarded as a strategy to ensure quality and should preferably reside as a standard component within the hospitals’ existing quality management [25]. It is recommendable to utilise data captured pursuant to the new Infection Protection Act for surveillance of resistant microorganism or antiinfective drug use (IfSG §23 Abs. 4) as well as selected data on infection management provided by external quality assurance sources. Additional quality indicators for local use should be selected and applied regularly. This allows to evaluate and document whether ABS aims can be met [171]. Owing to the different structures and organisation of hospitals, ABS measures must be evaluated locally and if need be adjusted accordingly [2, 22].

Ideally, indicators ought to be evidence-based, i.e. guideline-derived, and ought to be supported by a formal consensus process in regard of their relevance and practicability; last but not least, they ought to be also put to the practical test. Indicators have been developed for community-acquired pneumonia and urinary tract infections. However, numerous suggestions have been put forward for indicators whose evidence base is rather small and whose relevance and practicability rests on consensus alone. In Germany, there are catalogues of quality indicators for instance for the Helios Hospital Group (“Initiative of Quality Medicine”) or for the Rhön, Sana und Asklepios Hospital Group (“Quality Hospitals”). They also exist for mandatory external health care quality assurance concepts, for whose development and implementation the German National Institute for Quality Measurement in Health Care (Bundesgeschäftsstelle Qualitätssicherung gGmbH; BQS) till 2009, and since then the AQUA Insitut (the Institute for Applied Quality Improvement and Research in Health Care GmBH) was commissioned by the Federal Joint Committee (Gemeinsamer Bundesausschuss; G-BA). However, only few quality indicators have been set for measurement of antibiotic prescribing of which some are already at goal (e.g. for community-acquired pneumonia, antibiotic prophylaxis for obstetric and gynaecological indications, femur fracture, as well as hip and knee endoprosthesis). Individual more or less plausible and consented catalogues of structural indicators are available outside Germany [65, 173, 174]. A lot of experience was especially gained in France. Process quality indicators, respective pneumonia and surgical prophylaxis, are available in multiple countries (e.g. http://www.qualitymeasures.ahrq.gov or http://www.jointcommission.org or http://www.ic.nhs.uk).

The guideline committee in collaboration with the ABS Expert Network (http://www.antibiotic-stewardship.de) and the University Hospital of Freiburg established a catalogue of consensus structural and process ABS quality indicators in a multistage procedure including Delphi survey. The catalogue should facilitate external and internal quality assurance. Clinical, ecological (resistance) and economical (cost, cost-effectiveness) relevance as well as the presumed practicability were assessed separately in several categories. In analogy with the so-called QUALIFY process [175], a provisional list of potentially suitable structural and process indicators was drafted. It was based on the draft of the Guideline itself, the current literature [1, 6, 25, 86, 105, 171, 176–189], including documents and experience with the former ESAC Group (http://www.esac.ua.ac.be) [190] and the former ABS International Group, an initiative of 9 EU member states for the improved use of antiinfectives (http://www.abs-international.eu) [191]. Based on the results of a workshop (15 participants) held at the ABS Expert Network meeting in November 2011 in Freiburg, a later questionnaire survey (Delphi methods, *n* = 75 ABS experts, i.e. advanced members of the ABS training programme of varying professional background, incl. pharmacy and microbiology) and a further workshop, of the initial 99 potential indicators 67 were put forward for discussion and 21 structure and 21 process indicators subsequently selected as presumably most suitable (see Tables 7, 8 in the Appendix) [434]. Suitable indicators on the quality of antimicrobial prescribing (process indicator) and structure (structure indicator) taken from this list (Tables 7, 8 in the Appendix) ought to be set and used in every ABS programme. At least three indicators on structure and process quality each should be determined regularly.

### Supplemental ABS strategies

There are various strategies or measures that can supplement and complement the core ABS activities described here. They can play a pivotal role in further improvement of outcomes of antibiotic stewardship programmes. Supplemental ABS strategies include special programmes and recommendations for therapy optimisation, special rules in reporting microbiology results, rules on the management of patients with multidrug-resistant pathogens (MDR) or *C. difficile* as well as computerised support systems. Evidence for their effectiveness varies. Their implementation partly depends on the hospital’s infrastructure, e.g. with computerised expert systems and information technology or the possibility for rapid measurement of antibiotic levels in serum.

#### Special programmes for treatment optimisation

As a rule, programmes for therapy optimisation such as de-escalation (streamlining) strategies, interventions to control duration of treatment, switch to oral administration and optimisation of antimicrobial dosing are carried out at the ward or patient level (also called “point-of-care interventions”). These are relatively focussed interventions that can be highly effective in improving the quality of antimicrobial prescribing. They are usually elements of proactive audit of antiinfective use or chart review, implementation can, however, also be computerised. [2, 22, 25].

##### De-escalation

**The guideline development group recommends:**

A key aspect of supplemental measures is to streamline treatment after initial empirical broad-spectrum therapy and conversion from empirical to targeted therapy. This ought to be done based on clinical criteria as well as microbiology results or other diagnostic findings. De-escalation measures ought to be preferably performed at the patient level in the context of antiinfective point-of-care chart reviews and proactive audits of antiinfective drug use **(B)**. Programmes promoting antiinfective de-escalation are expected to, by reducing antibiotic load, impact beneficially on the emergence of resistance, the prevention of secondary infections, cost levels and adverse drug reactions **(B)**.

De-escalation proposes to simplify treatment, i.e. monotherapy rather than combination therapy, targeted (narrow spectrum) rather than untargeted broad-spectrum therapy, discontinuation of empiric treatment if diagnosis is uncertain [192, 193]. There are insufficient data to favour combination therapy over monotherapy in the routine management of ventilator-associated pneumonia. In addition, combination therapy did not show a benefit with regard to decreasing superinfection rates or the emergence of resistant pathogens [6, 194–197]. In a meta-analysis of eight randomised, controlled studies on patients with different infections β-lactam/aminoglycoside combination therapy did not impact favourably on the emergence of resistance. Fewer superinfections were observed with monotherapy (OR 0.62; 95 % CI, 0.42–0.93) than with combination therapy [198]. Furthermore, combination with aminoglycosides is associated with a higher incidence of nephro- and ototoxicity [199–201]. Thus, de-escalation to monotherapy is recommended based on type of infection and microbial culture results [202]. Combination therapy is only recommended for selected indications.

Observational studies on general wards and intensive care units show that 20–60 % antibiotic treatments could be adjusted based on microbial findings alone [193, 203–205]. Pharmacists and infectious diseases physicians assessed combination therapy as being unnecessary in 50 % of cases, measurable effects of de-escalation were reduced length of hospital stay and high-cost savings [206–208]. In an intervention, a computer programme identified combination antibiotic therapy across the hospital, which was then evaluated by a pharmacist or infectious diseases physician for adequacy. 98 % of combination therapy was found to be redundant. The implementation led to considerable net savings [209].

The effect of de-escalation, or treatment adjustment based on microbial culture results and/or clinical criteria was well demonstrated in at least one multicenter clinical study of patients in intensive care. Patients suspected of having developed ventilator-associated pneumonia receiving treatment in 31 French intensive care units were switched to targeted treatment based on culture and sensitivity results of pathogens obtained by bronchoalveolar lavage or endotracheal aspiration. Patients who received treatment based on bronchoalveolar lavage culture results had significantly more antibiotic-free days (5 vs 2), significant 10 % lower mortality at day 14 and decreased sepsis-related organ failure at day 3 and 7 [100, 103]. The authors related this to the fact that invasive bronchoscopy allows to differentiate better between pulmonary infection and colonisation, and that antibiotic treatment can be discontinued earlier given negative cultures from bronchoalveolar lavage. In a randomised controlled trial conducted in an intensive care unit in North America, the course of antibiotic therapy of pneumonia was shortened by 2 days, based on clinical criteria, without negative impact on mortality [210]. Numerous other investigations confirm these observations and show that adjusting therapy is usually possible after 48–72 h [211–214].

##### Duration of treatment 

**The guideline development group recommends:**

It is possible to shorten the duration of antiinfective treatment for many indications (e.g. perioperative antibiotic prophylaxis) and this is recommended wherever backed by good studies and evidence. The ABS team should utilise local guidelines and antiinfective point-of-care chart reviews to draw attention to the excessive duration of treatment frequently encountered in practice. The ABS team should define the duration of treatment recommended as a rule, since this is expected to impact substantially on antiinfective drug use, side effects and costs **(A)**. Use of biomarkers such as Procalcitonin may be useful for controlling the duration of treatment in cases where there is clinical uncertainty. As a result, the number of days of antibiotic therapy can be reduced and under certain circumstances costs can be cut **(C)**.

A frequently encountered problem in regard to antibiotic therapy is the duration of antimicrobial treatment often being too long. Large-scale studies in the USA, in European countries and elsewhere have repeatedly demonstrated prolonged duration of perioperative antibiotic prophylaxis—in 50 % or more cases perioperative antibiotic prophylaxis was administered for longer than 24 h [85, 86, 215]. This unnecessarily increases selective pressure for resistance to emerge [102, 216–219].

German recommendations with S3 Guideline level, e.g. on community-acquired and hospital-acquired pneumonia or uncomplicated community-acquired urinary tract infections, give explicit, evidence-based recommendations on duration of treatment (http://www.awmf.org). The results of the studies on which the recommendations on pneumonia are based demonstrate convincingly that the mean duration of treatment can be reduced without negative impact on clinical outcome and mortality [102, 220, 221]. An important trial is a prospective randomised double-blind study of patients with ventilator-associated pneumonia undertaken in 51 French intensive care units. It was demonstrated that 8-day treatment had no disadvantage for these patients as compared to 15 days. Shorter treatment duration was associated with emergence of fewer multidrug-resistant pathogens (−20 %) [101, 102]. These findings and the results of other similar studies have had a decisive influence on the assessment and conclusions reached in recent meta-analyses [222, 223]. Other good scientific studies on urinary tract infection show similar results [224]. When implementing ABS programmes, treatment orders for patients with community-acquired pneumonia should for instance be linked to a note “no longer than 5–7 days” to remind users to undertake an individual clinical reassessment of further treatment beyond this point in time. This question can also be addressed in proactive audits of antiinfective drug use [225, 226].

Biomarkers can also be useful to guide duration of therapy. Corresponding studies are available on use of Procalcitonin particularly in the management of patients with respiratory tract infections, whereby treatment duration in the control arm does not correspond in some cases to today’s standards. Various systematic reviews and meta-analyses are available on Procalcitonin [227, 228]. Determination of Procalcitonin levels can influence the density of antibiotic treatment in intensive care units: in a prospective study antibiotic treatment was reduced by 23 %, in other studies this effect remains, whereby the cost-effectiveness is unclear and there seems to be no impact on mortality [229–235].

Numerous studies have aimed to improve adherence to guidelines on perioperative antibiotic prophylaxis, especially with regard to duration. Training programmes, local guidelines and checklists in the operating room with and without use of computer-based information technology were most often applied. For various reasons the results are not always satisfactory and comparable, depending on intervention and speciality field [128, 129, 158, 188, 236–242]. Some examples of successful outcome are the significant reduction of treatment duration from 2.4 to 1.6 days (Japan), the 15 % reduction in the amount of antibiotics prescribed for perioperative antibiotic prophylaxis (Germany), the increase in the proportion of guideline-adherent prophylaxis of no longer than 24 h duration from 3 to 66 % (Taiwan) and the reduction of prolonged prophylaxis >24 h from 21 to 8 % (Netherlands). A time-series analysis of 13 hospitals in the Netherlands demonstrated convincingly that targeted training programmes result in a decrease of perioperative antimicrobial drug use from 121 to 99 DDD (defined daily doses)/100 procedures, and in a cost reduction of 25 % per procedure [188]. If available, electronic prescribing systems can be used for automatic stop orders to reduce the proportion of patients with prolonged prophylaxis. In a US study, by computer-based order intervention, the proportion of patients who had prophylaxis discontinued in the appropriate time frame increased by 17 %, compared to no change without intervention [243].

##### Parenteral-to-oral conversion

**The guideline development group recommends:**

If sufficient bioavailability is assured, and if the patient’s condition allows, therapy should be switched from parenteral to oral antibiotic application **(A)**. This measure reduces the length of hospital stay and the risk of line-related adverse events. Furthermore, it leads to a reduction in the total cost of treatment. Implementation of programmes allowing parenteral-to-oral conversion of antimicrobial agents at the institutional level ought to be facilitated by developing clinical criteria and through explicit designation in institutional guidelines or clinical pathways **(B)**.

Critically ill patients suffering from an infection, initially receive parenteral antibiotics. Stabilised patients as well as patients with a less serious illness can be given oral agents with good bioavailability as long as there are no contraindications (e.g. disorders of gastrointestinal resorption or dysphagia) (Table 6). Conversion to oral administration has numerous advantages. Mobility is improved, patients can be discharged earlier, the risk of adverse line-related events is smaller, and the amount of nursing time required is usually reduced. Switch to oral antibiotics has been well investigated for certain indications, and recommendations are made in many guidelines, e.g. the German guideline for treatment of community-acquired pneumonia [6, 244–246]. Safety of switch to oral administration was evaluated in at least one meta-analysis and several partly multi-centre randomised controlled clinical trials. It was demonstrated, that duration of parenteral treatment and length of stay can be reduced by approximately 2–3 days without increasing mortality [61, 247–255]. In a prospective quasi-experimental observational study involving around 200 pneumonia patients, almost 70 % of the patients could be switched to oral antibiotics at day 3, and a further 20 % between day 4 and 7. Similar experience was made in a series of other observational studies [39, 76, 111, 256, 257]. Safety was also investigated with respect to study endpoint hospital readmission [258, 259].

**Table 6:** Substances with good-to-excellent bioavailability

• Fluoroquinolones (without norfloxacin)	
• Cotrimoxazole	
• Doxycycline	
• Metronidazole	
• Linezolid	
• Rifampicin	
• Fluconazole	

With the exception of endocarditis and meningitis, timely switch to oral antibiotic administration can also be reasonable for pyelonephritis, for skin and soft tissue infections, febrile neutropenia, infantile osteomyelitis/purulent arthritis [252, 260–267]. Systematic review of good clinical studies partially performed in Europe shows that switch to oral antibiotic administration for another 7–11 days is already possible at day 3 of parenteral therapy for treatment of infantile pyelonephritis without a higher incidence of renal damage or other complications [268–271]. Other studies have documented substantial cost savings from early shift to oral antibiotics and as a consequence earlier discharge from hospital [64, 272–274]. Some of these investigations were conducted by hospital pharmacists themselves [39, 275]. Prospective observational studies investigating early switch to oral antibiotics have demonstrated that sustainability can be achieved by checklists, clinical pathways defining criteria for early conversion to oral therapy and its implementation supported by hospital pharmacists [258, 276–280].

##### Dose optimisation 

**The guideline development group recommends:**

Adequate adjustment and optimisation of the dose and dosing interval is essential for effective, safe and responsible administration of antiinfective therapy, and an important part of ABS programmes. Besides individual patient factors, optimal dosing of antiinfectives should take into account, the nature and severity of illness, the causative microorganism, concomitant medications, as well as the pharmacokinetics and pharmacodynamics of the agents prescribed. Strategies to optimise dosing in ABS programmes should include assessment of organ function for drug dose adjustment in order to avoid adverse drug events and unwanted drug interactions **(A)**.

Furthermore, optimising the dosing interval and duration of infusion is recommended in particular in critically ill patients, best by employing a therapeutic drug monitoring (TDM) scheme; appropriate consented local institutional guidelines should be available and up to date **(B)**.

Evaluation of ABS programmes showed that one-third of all interventions was in regard to dose optimisation of antimicrobial treatment. This was demonstrated in many retrospective studies which provided evidence of inappropriate choice of agent and inadequate dosing [281–287]. As with all medication, antiinfective dosing requires individual review and adjustment. If need be, dose and dosing interval must be adjusted, whereby age, weight, gender, hepatic and renal function, underlying and concomitant disease as well as co-medication must be considered. Dosing is largely determined by pathogen susceptibility, the location and severity of infection [288]. Dose optimisation programmes have been implemented by pharmacists and infectious diseases physicians with similar success and can also be cost effective [37, 289–297].

Pharmacokinetic and pharmacodynamic (PK/PD) properties are important to achieve optimal antiinfective dose levels [298, 299]. Emergence of resistance can be promoted by using incorrect, low dosages of antibiotics with low-resistance barrier [216]. Therefore, strategies to avoid incorrect drug dosage or suboptimal dispensing appear useful, at least in critical areas such as intensive care units, despite uncertain evidence for a clinical benefit [101, 300–305]. Important strategies are optimisation of dosing intervals (e.g. higher concentrations of aminoglycoside with an extended dosing interval) [306] and prolonged infusions of beta-lactams especially in presence of critical illness or multidrug-resistant microorganisms [307]. In this setting, therapeutic drug monitoring (TDM) can improve antibiotic dosing [308–310].

By using TDM, the proportion of patients with serum piperacillin concentrations within therapeutic range increased from 50 to 75 % in a 30-bed intensive care unit in a French teaching hospital [311]. A much cited, multi-centre study from the Netherlands conducted in medical and surgical wards of four hospitals was able to show that active TDM-guided dosing of aminoglycosides and dosing recommendations by clinical pharmacists can significantly shorten length of stay by approximately 6 days and reduce incidence of nephrotoxicity from 13 to 3 %. A highly detailed cost-effectiveness analysis showed overall cost savings of 30 % [312]. Similar results were reported from France [313, 314]. The benefits of single-dose aminoglycoside administration compared to multiple-dose and extended-interval aminoglycoside dosage regimens can be utilised to minimise nephrotoxicity in children [38, 315, 316].

PK/PD analyses show that with beta-lactam antibiotics the duration of time that drug levels exceed the MIC of the pathogen at the site of infection is important to achieve better treatment outcomes (time-dependent killing). Beta-lactams with short half lives (<2 h) should actually be given as extended or continuous infusion, which requires drug stability at room temperature [317, 318]. An older meta-analysis [319, 320] suggests that clinical outcome in continuous intravenous infusion of suitable beta-lactams seems to be superior to intermittent infusion of the same daily dose. More recent systematic reviews investigating continuous infusion have not confirmed superiority [310, 321], whereby different daily doses were compared, though in certain studies very good effects were observed. McKinnon et al. [322] for instance showed in a prospective randomised investigation in critically ill patients with bacteremia that continuous infusion ceftazidime or cefepime is superior to intermittent short-term regimen: clinical improvement and mortality differed significantly. In a randomised study of bacterial meningitis, where cefotaxime was administered in the first 24 h by continuous infusion versus intermittent bolus, a benefit was seen in favour of continuous infusion in terms of lower mortality [323]. Treatment success can be achieved by the drug concentration being 40–60 % of the time in the 2- to 4-fold range of the MIC. Drug concentrations remaining above the MIC 100 % of the time is not necessary. An intermittent dosing strategy with longer duration of infusion can thus suffice to guarantee optimal outcome [324]. A retrospective multicenter study showed that increasing length of infusion (4 h) gives better clinical results for intermittent dosing of piperacillin/tazobactam. Mortality was reduced from 18 to 10 % (*p* = 0.02) [325]. A similar (before and after) observational study (larger number of cases) with cefepime, piperacillin/tazobactam and meropenem could not reproduce these positive results [326]. In yet a further prospective investigation conducted across Australia and Hongkong with 2 × 30 patients (in addition to piperacillin/tazobactam, ticarcillin/clavulanate and meropenem were permitted), PK parameters were defined as primary endpoint. The clinical results are of interest nevertheless: clinical cure was observed in 70 vs 43 % (continuous vs. bolus) (*p* < 0.01), and survival in the two arms was 90 vs 80 % [327]. In a Czech study, 2 × 120 patients (intensive care, medium APACHE-II-Score >20, high incidence of Klebsiella infection) were randomised in continuous infusion group and bolus group. A high loading dose was given in both arms. Clinical cure (83 vs. 75 %, *p* = 0.18) and microbiological success rate (91 vs. 78 %, *p* = 0.02) were better with continuous infusion. However, a very high loading dose of meropenem was given in both arms (4 × 1 g over 6 h vs. 3 × 2 g over 30 min) [328]. Additional findings in the continuous infusion group were shorter ICU stay (10 vs. 12 days), shorter duration of therapy (7 vs. 8 days) and (as to be expected from the study design) lower total dose of meropenem. Concerning mortality, no significant statistical difference (hospital, ITT population) was detected (17 vs. 23 %).

##### Scheduled switch of antimicrobials

**The guideline development group recommends:**

So-called “Cycling” programmes, which involve periodically removing a specific antimicrobial drug or an antimicrobial drug class as the standard recommended therapy and later reintroducing it (periodic scheduled rotation), are not suitable as a strategy to reverse critical emergence of resistance or to control nosocomial outbreaks with multiple resistant pathogens and, as such, should not be used as a strategy to do so **(A)**.

Strategic rotation of specific antimicrobial drugs or antimicrobial drug classes ought to be undertaken to limit the selective pressure and to achieve a reduction of infectious microorganisms or microorganisms displaying specific resistance properties for a certain time **(B)**. There is evidence to suggest that a balanced use of different antimicrobial drugs or antimicrobial drug classes (so-called “mixing”) can minimise the emergence of resistance. In both cases, routine surveillance of antimicrobial drug use and resistance should be performed **(A)**.

Repeated rotation of different antimicrobials or antimicrobial classes (e.g. cephalosporins, fluoroquinolones, penicillins, carbapenems) for empiric therapy of acute infection within an institution or specific unit—so-called “Cycling”—was designed to limit selective pressure and thus prevent development of resistance toward frequently prescribed antimicrobials/antimicrobial classes. Published experience with cycling strategies, mainly on aminoglycosides, is a few decades old, and, in view of the minor share of aminoglycosides used, of little interest in clinical routine today. Neither are the studies consistently successful in showing a detectable effect in minimising resistance [6, 329, 330]. In many cases, implementation was problematic, with up to 50 % of the patients in cycling programmes receiving “off cycle” antimicrobials, i.e. not receiving per protocol antimicrobial treatment [6].

More recent studies on cycling of broad-spectrum beta-lactams show little improvement in methodology or results [331–337]. Neither do mathematical models of antimicrobial cycling demonstrate benefit in respect of avoiding resistance. Mathematical modelling suggests that antibiotic cycling strategies, prompting simultaneous diversity of antimicrobials/antimicrobial classes, perform better than temporary dominance of a single antimicrobial agent/antimicrobial class [338–340]. Several clinical trials confirm this concept [341–344]. Within the scope of a Spanish prospective intervention study on ventilator-associated pneumonia in an interdisciplinary intensive care unit, it could indeed be demonstrated that, compared with two strategies with heterogenous use of cephalosporins, penicillins, carbapenems and fluoroquinolones, cycling led to a significant increase in resistant nosocomial pneumonia pathogens [345]. Thus, with respect to antimicrobial classes, attention should be paid to use of balanced, guideline-adherent therapy. Above all, excessive use of cephalosporins and fluoroquinolones should be avoided. If surveillance data of cephalosporins and fluoroquinolones point to excessive consumption, a strategic class switch in preference of penicillins should be undertaken. There are several new publications on this topic. Following an educational campaign and subsequent introduction of restrictions, the policy “Reduction of routine use of ceftriaxone and ciprofloxacin in favour of aminopenicillins” was successfully implemented in a Scottish hospital. The endpoints observed were change in the incidence of hospital-acquired MRSA and ESBL-positive infections (without special screening) as well as *C.difficile* rates. As result of the new policy, consumption of ceftriaxone reduced by 95 % and that of ciprofloxacin by 73 % (comparison of the first and final 6 months of the study). At the same time, hospital-acquisition rates for *C.difficile* reduced by 77 %, MRSA by 25 % and ESBL cases by 17 %. The intervention had a sustained effect (up to 3 years later) [346]. Other observational studies on strategic antimicrobial substitution of cephalosporins in favour of penicillins conducted in China, Greece and India seem to confirm a decline in ESBL cases, however, cannot prove it [347–350].

#### Special rules for communication of microbiology results

**The guideline development group recommends:**

The quality of microbiology diagnostics depends crucially on compliance with guidelines on procedures in the preanalytical phase. Expert consensus recommends that any deviations from protocol ought to be reported and the reasons for rejecting the samples stated **(B)**.

Technical progress and up-to-date molecular diagnostic methods for rapid pathogen detection should be used if they improve the quality of care and/or substantially improve identification and epidemiologic investigation of local outbreaks **(A)**.

Positive blood culture findings, interim microscopic findings, results of rapid testing and rapid susceptibility testing should be delivered promptly to the attending physician **(A)**.

Antibiograms ought to adhere to local guidelines with respect to antimicrobial use and diagnostic findings, be presented selectively in agreement with the ABS team, and, if need be, include relevant interpretative comments. This procedure aids selection of a targeted, guideline-based antibiotic therapy **(B)**.

The microbiology laboratory is responsible for the timely identification of trends in antimicrobial resistance and prompt communication of observations to the ABS team and the physicians responsible for infection control **(A)**. This way, the clinical and epidemiological significance of the observations can be defined at an early stage.

The microbiology laboratory plays a crucial role in achieving the objectives of an antimicrobial stewardship programme by providing timely identification of relevant pathogens, selective antibiograms and active communication of diagnostic results and their interpretation, including interim reports. Microbiology diagnostics and susceptibility testing and reporting should be based on latest national [e.g. quality standards of microbiology and infectious diseases (MiQ) for Gemany] and international quality standards (http://www.eucast.org), should address the specific requirements of the requesting physician and live up to the hospital’s obligation to provide medical care. A meaningful microbiological diagnostic requires optimal sample quality, storage, and timely transport of samples to the laboratory. The transport time of urine samples stored at room temperature should not exceed 2 h, since delays in transport of samples to the laboratory may increase pathogen growth and produce false-positive test results [351, 352]. Samples that deviate from guideline-adherent pre-analytics ought to be reported or ought to lead to the implementation of rejection criteria. Criteria ought to be defined for all common samples—e.g. sputum, urine, stool and swabs—to avoid unnecessary antimicrobial therapy. It is for instance recommended that purulent sputum with more than 25 squamous epithelial cells per field ought not to be further screened; rather, findings ought to be reported and the sputum sample discarded [353–355]. Microbiological diagnostic of good-quality sputum samples can provide valuable information on the pathogen involved. Older studies investigating patients with pneumonia were able to show that high-quality sputum samples resulted in targeted antimicrobial monotherapy more often [356, 357] than inadequate sputum or no sputum culture. Reporting is also of importance on samples from non-implanted foreign bodies (drains, urinary catheters, venous catheters, tracheal cannula, wound sponge, etc.) or where low sample volume impairs sensitivity.

Automated MIC-based antibiograms and integration of molecular diagnostic assays such as PCR, PNA-FISH or MALDI-TOF into microbiology diagnostic can shorten time to pathogen identification and reporting of results [358]. Several prospective, randomised clinical studies on diagnostic of blood cultures and respiratory samples in pneumonia have shown that more rapid detection of pathogens by a few days resulted in earlier targeted antibiotic therapy. Thus, duration of empirical therapy, duration of ventilation and length of hospital stay were reduced by a few days and mortality decreased variably [36, 359–366]. More recent prospective investigations on use of rapid *Legionella* urinary antigen test in pneumonia patients have shown that it is a useful tool with which targeted antimicrobial treatment can be provided more frequently. This was less conclusively demonstrated for pneumococcal antigen in urine [367, 368]. However, carefully developed and implemented algorithms are necessary to maintain the potential benefit. At least two controlled before-and-after studies have shown that antibiotic therapy is only modified and adjusted to microbiological findings, i.e. blood culture results, if these are communicated personally to the treating physician or the culture results are documented in the patient’s medical chart [369–371].

The guideline development group recommends the use of selective reporting of susceptibility testing results with respect to choice and number of antimicrobial agents depending on pathogen, local susceptibility data and existing therapy guidelines, with the objective of supporting guideline-adherent antibiotic therapy [1, 6, 372]. Two methodologically different interrupted time-series analyses indicate that selective antibiotic susceptibility reporting can influence prescribing behaviour [373, 374]. Additional reporting, providing information on major resistance mechanisms, on contamination or colonisation according to pathogen and pathogen quantity, or information on diagnostic and therapeutic guidelines can support ABS measures. However, the effect of the mode of reporting microbiology results on prescribing behaviour is not well studied.

In case of unprecedented or critical levels of bacterial resistance, the microbiology laboratory should identify the cause by molecular biological methods as well as characterisation of clonal variants by typing, in particular during outbreaks and in this case especially with the assistance of reference laboratories. Depending on test results, targeted ABS strategies should be seized by the ABS team in accordance with appropriate hygiene interventions, implemented by the infection control team.

#### Special rules for management of patients with multidrug-resistant microorganisms and *C. difficile*

**The guideline development group recommends:**

ABS strategies should be used to prevent infection with *C. difficile***(A)**. Restricting use of certain antimicrobial drugs or substitution of antimicrobial drug classes (e.g. penicillin for cephalosporins or fluoroquinolones) can considerably reduce the incidence of *C.difficile* infection. Infection prevention and control strategies are frequently also applied at the same time; however, they have less impact on the *C. difficile* incidence than in the epidemiology of MRSA or VRE.

Targeted ABS strategies are to varying degrees also effective in reducing multidrug resistant Gram-negative bacteria, particularly ESBL-producing microorganisms, MRSA and VRE, and ought to be specifically applied here too **(B)**. In case of high prevalence of multidrug resistant microorganisms, recommendations on diagnostic tests, evaluation of findings and treatment, as well as infection control management should be coordinated immediately and disseminated locally **(A)**.

Routine surveillance of antimicrobial consumption and antimicrobial susceptibility data should be performed **(A)** to avoid indiscriminate compensatory use of other antimicrobial drug classes, since this can promote the unintentional and uncontrolled emergence of resistance.

The importance of appropriate ABS strategies for the management of patients with multidrug-resistant microorganisms and *C.difficile* is well documented in several systematic reviews and corresponding studies especially for *C.difficile* [2, 8, 22, 25, 375]. Pretreatment especially with third-generation cephalosporins and fluoroquinolones presents a risk factor for *C. difficile* infection, as well as for the increase in ESBL-producing Gram-negative microorganisms, MRSA and VRE [376, 377].

In before-and-after studies restriction of some types of antimicrobials, particularly third-generation cephalosporin and fluoroquinolones, but also macrolides and clindamycin, resulted in a 50 % or higher reduction in the incidence of *C. difficile*-associated disease. Frequently, interventions were accompanied by general infection control strategies. However, even in controlling outbreaks, infection control practices do not always appear to be sufficiently successful, as shown by the data collected retrospectively in a methodologically robust time-series analysis. It was not until restriction of cephalosporins, macrolides and clindamycin was imposed 6 months later that the incidence of *C. difficile*-associated disease decreased by 60 %. Utilisation of broad-spectrum penicillin subsequently increased compensatorily without repeated emergence of the epidemic *C.difficile* strain [73]. An investigation carried out over several years in a geriatric unit showed that the incidence of *C. difficile* diarrhoea was strongly associated with the density of cefotaxime use [378]. More recent time-series analyses over a period of 12–24 months confirm that substituting cephalosporins and fluoroquinolones for penicillins can lead to a decrease in the incidence of *C.difficile*-associated disease. Cephalosporin and fluoroquinolone use decreased considerably by more than 22–50 % [144, 146].

It is possible to influence the incidence of ESBL-positive isolates by implementing strategies to control antibiotic consumption. Various methodologically different but demanding studies on restriction of cephalosporin use showed a decrease in the rate of infection and colonisation with ESBL-producing microorganisms [349, 350, 379–381]. However, in respect to sustained minimisation of resistance, the effects of a policy for targeted restriction of antimicrobials are less conclusive [124, 382] and sometimes even contradictory [383–387]. Particularly, unplanned increased consumption of alternative agents, in setting of restriction, can rapidly impact negatively on the resistance situation [135, 388, 389]. The effect of restriction of third-generation cephalosporins, vancomycin and/or fluoroquinolones varied in regard to VRE and MRSA [117, 383–385, 390, 391]. For instance, a short-term effect of restriction of vancomycin and cephalosporins on gastrointestinal colonisation with VRE (decrease from 47 to 15 %) was observed [392]. According to long-term observations, VRE eventually increased again [393]. Infection control practices seem to have a stronger sustained effect in this regard, and ABS strategies alone may not suffice.

In case of high incidence of multidrug-resistant microorganisms and cumulative outbreaks, appropriate recommendations on diagnostic tests, evaluation of findings and treatment, as well as infection control management must be coordinated immediately and disseminated locally. There is generally great uncertainty about optimal treatment [394]. In certain circumstances, use of unconventional antimicrobials or of conventional antimicrobials in unusual dose and combination may become necessary. In this situation, it is indispensible for the ABS team to draft appropriate guidance and recommendations in collaboration with the microbiology laboratory, to allow optimal treatment outcome and not to promote further spread of multidrug-resistant microorganisms through inadequate antibiotic use.

#### Computerised information technology

**The guideline development group recommends:**

The ABS team should be supported by novel information and communication technology in the implementation of ABS programmes. Local treatment guidelines, the antiinfective formulary, and other ABS documents should be available electronically **(A)**.

Electronic prescribing tools with and without linkage to electronic preauthorisation solutions to ABS documents or to active communication of information using computerised reminders to the prescriber should be used to improve the use of antiinfectives in the interests of patient safety **(A)**. They ought to be used to reduce consumption and/or costs **(B)**.

Computerised decision support systems that are integrated into the hospital’s internal information system can, by utilising electronic medical records, help evaluate and optimise the indication for antiinfective therapy, drug selection and dosing **(C)**.

To implement computerised ABS measures, the ABS team must have hospital-wide access rights to electronic medical records (with due respect to data protection).

The ABS team should be supported by novel computer-based information and communication technology through the provision of hospital-wide availability of ABS documents (antiinfective formularies, guidelines, treatment pathways). Development and application of electronic computerised decision support systems (CDSS) is to be encouraged. To utilise these systems optimally, it is useful to link them to an electronic patient record/chart or/and a patient-based computer physician order entry system (CPOE). In spite of the tremendous advances that have been made in the development of these systems in the hospital sector, availability varies in German and Austrian hospitals. Software designed specifically for ABS purposes hardly exists at all. Systematic reviews evaluating the impact of CDSSs specifically in the field of ABS (studies to 2007) found them to be of limited benefit [395, 396].

Electronic hospital information systems are used to varying extent in most hospitals in Germany. The system gives the treating physician access to patient-related and treatment-relevant data. In the interests of patient safety, and taking account of data protection, the ABS team should also have access to these data (antiinfectives, and microbiological laboratory results). Furthermore, electronic hospital-wide surveillance data on pathogens and antiinfective consumption should be available to the ABS team at all times. The ABS team should receive support on use and layout of computerised information systems from experts [397–401].

Besides automated electronic alerts/short consultations and computerised decision support systems, in future, electronic prescribing systems, i.e. patient-based computer physician order entry systems (CPOE) with and without approval and reminder/alert functions will be particularly important for interventional ABS activities. As far as prescribing quality is concerned, these systems offer many advantages over paper-based medication orders with regard to legibility, completeness, fast delivery of information and possibly to approval or reminder/alert functions [402]; however, they are not as yet standard practice in German-speaking countries. By using CPOE systems, the rate of medication ordering errors can be reduced considerably (dosing, interactions, allergies) [403–407]. On the other hand, the extent to which these systems influence resistance or clinical outcomes and mortality is inconclusive [408–413]. In addition, CPOE systems could facilitate audits of antimicrobial prescribing through timely provision of patient-related, cross-unit and cross-departmental antimicrobial prescribing data [414, 415]. In a randomised controlled trial conducted in the USA, an ABS team [infectious diseases physician (50 % FTE), clinical pharmacist (80 % FTE)] reviewed antibiotic prescribing based on a list of alerts generated by an electronic decision support system. Among others, the following selection criteria were used: intravenous antimicrobial application in spite of good oral bioavailability, unnecessary combination therapy, and antibiogram discordant therapy. Compared with the control arm, USD 38 were saved per day per patient (around 16 % of total antibiotic costs) and 1 h of work a day with automated system alerts [416, 417]. A reduction in rates of antimicrobial use can be achieved by integrating an antimicrobial approval system into electronic prescribing [75, 243, 408, 409]. Web-based reviews of guideline-adherent prescribing of third-generation cephalosporins resulted in a significant 50 % reduction that was sustained over 15 months [75]. In another study, vancomycin use required an indication (by drop-down menu or free text) at initial electronic ordering and after 72 h. Treatment was stopped automatically if no indication was entered both times. As a result, initial vancomycin orders per physician were reduced significantly by 29 %, and 36 % fewer renewal orders were written after 72 h. The amount of days of vancomycin therapy decreased by 36 %. However, no information was provided on clinical outcome [418]. Electronic prescribing systems can contribute to reduce antibiotic expenses, if information on antibiotic cost/hospital day is provided while the order is placed [419]. This seems to be more effective than automated feedback to prescribers on antibiotic expenditure in comparison to others [420].

Electronic documentation of the timing of perioperative antibiotic prophylaxis with provider-specific feedback (confidentially to the anaesthetist) on the rate of prophylaxis given too early or too late appeared to be effective. At the time the intervention was initiated 69 % of the patients received antibiotics within 60 min of incision, and 92 % a year later [421]. Similar good-to-very good results were reported for the use of so-called electronic “Real-time Alerts” in improving timing of perioperative prophylaxis (>99 % of perioperative prophylaxis in one study). An electronic alert system was implemented after 4 h operating time to remind surgical teams to redose perioperative prophylactic antibiotics. The intervention was effective: 68 % of patients with cardiac surgery in the reminder group received intraoperative redosing versus only 40 % in the control group. The rate of surgical site infection was similar in both groups (4 und 6 %), but lower than in the pre-study period (10 %) [422, 423].

Several studies on electronic expert systems were conducted in a hospital in Salt Lake City (1986–2001). Implementation in many German hospitals, however, is difficult on account of inadequate technical prerequisites. These systems provide clinical decision support in the form of detailed therapy recommendations and alerts based on interlinked data such as allergies, laboratory results, microbiological findings, etc. Studies on this programme showed a significant reduction in the number of antibiotics prescribed, duration of treatment, costs, and adverse drug events [237, 424–431]. A comprehensive computerised system consisting of an antimicrobial guideline and approval system in regard to reserve antibiotics also proved highly effective in an Australian hospital. It facilitated turning around a trend towards greater use of reserve antibiotics and resulted in increasing conformity with guideline-recommended therapies [432]. Effects were less strong in other studies. In a European multi-centre study (incl. Denmark, Italy, Germany) a computerised decision support system that looked at local susceptibility data, showed a rate of “appropriate” empirical antibiotic treatment of 73 %, which was only negligibly better than that of the control group of 64 % [433]. A reduction of just under 11 % in antibiotic consumption was achieved in an Australian intensive care unit, while the proportion of inappropriate antimicrobial use declined by only 10 % [412].

## Figures and Tables

**Table 7:**
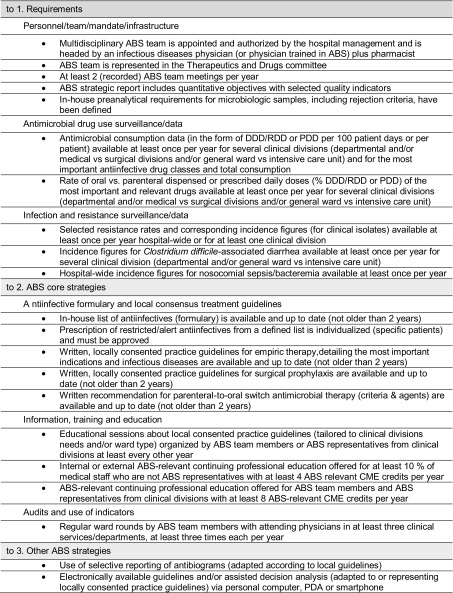
ABS structure indicators

**Table 8:**
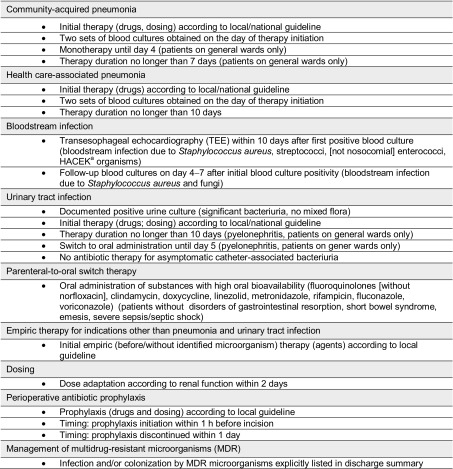
ABS process indicators

*TEE* transesophageal echocardiograph

^a^HACEK organisms are a group of Gram-negative pathogens that share an enhanced capacity to produce endocardial infections: *Haemophilus species* (*H. parainfluenzae, H. aphrophilus, H. paraphrophilus*), *Actinobacillus actinomycetemcomitans, Cardiobacterium hominis, Eikenella corrodens*, and *Kingella species*

## Appendix

See Tables 7, 8 (Sect. 2.2.4).
